# Heat Capacity of Indium or Gallium Sesqui-Chalcogenides

**DOI:** 10.3390/ma17020361

**Published:** 2024-01-11

**Authors:** Květoslav Růžička, Václav Pokorný, Jan Plutnar, Iva Plutnarová, Bing Wu, Zdeněk Sofer, David Sedmidubský

**Affiliations:** 1 Department of Physical Chemistry, Faculty of Chemical Engineering, University of Chemistry and Technology, Prague, Technická 5, 166 28 Prague, Czech Republic; ruzickak@vscht.cz (K.R.); pokorny@imc.cas.cz (V.P.); 2 Institute of Macromolecular Chemistry, Czech Academy of Sciences, Heyrovského Nám. 2, 162 06 Prague, Czech Republic; 3 Department of Inorganic Chemistry, Faculty of Chemical Technology, University of Chemistry and Technology, Prague, Technická 5, 166 28 Prague, Czech Republic; jan.plutnar@vscht.cz (J.P.); iva.plutnarova@vscht.cz (I.P.); bing1.wu@vscht.cz (B.W.); zdenek.sofer@vscht.cz (Z.S.)

**Keywords:** In, Ga, sesqui-chalcogenides, heat capacity, enthalpy, entropy, Gibbs energy

## Abstract

The chalcogenides of p-block elements constitute a significant category of materials with substantial potential for advancing the field of electronic and optoelectronic devices. This is attributed to their exceptional characteristics, including elevated carrier mobility and the ability to fine-tune band gaps through solid solution formation. These compounds exhibit diverse structures, encompassing both three-dimensional and two-dimensional configurations, the latter exemplified by the compound In_2_Se_3_. Sesqui-chalcogenides were synthesized through the direct reaction of highly pure elements within a quartz ampoule. Their single-phase composition was confirmed using X-ray diffraction, and the morphology and chemical composition were characterized using scanning electron microscopy. The compositions of all six materials were also confirmed using X-ray photoelectron spectroscopy and Raman spectroscopy. This investigation delves into the thermodynamic properties of indium and gallium sesqui-chalcogenides. It involves low-temperature heat capacity measurements to evaluate standard entropies and Tian–Calvet calorimetry to elucidate the temperature dependence of heat capacity beyond the reference temperature of 298.15 K, as well as the enthalpy of formation assessed from DFT calculations.

## 1. Introduction

In the past decade, materials for low-energy electronics have emerged as a focal point in current material research. The family of layered chalcogenides stands out as significant candidates for the advancement of innovative electronic and optoelectronic devices, owing to their exceptional properties such as high carrier mobility, a tunable band gap through solid solution formation, and their layered structure. Sesqui-chalcogenides of indium and gallium, denoted by the general formula M_2_Ch_3_, exhibit diverse polymorphic forms based on their composition and synthesis procedures. Gallium sulfide possesses a monoclinic structure, while gallium selenide and telluride adopt cubic structures. Indium sulfide takes on a tetragonal form, indium selenide features a hexagonal layered structure, and indium telluride has a cubic structure. The layered van der Waals structure of In_2_Se_3_ allows for its exfoliation down to a single-layer material [1,2].

Indium and gallium chalcogenides, particularly, hold promise for the construction of photodetectors and solar cell devices [3]; their high thermal stability is a great advantage for these applications. These materials have been synthesized in various forms, including bulk crystals from elemental sources, and thin films through chemical vapor deposition (CVD) and physical vapor deposition (PVD) methods, as well as exfoliation [4,5,6]. Layered indium(III) selenide, known for its semiconducting properties and ferroelectric characteristics, exhibits different structural polymorphs [7]. Nanostructures of such layered materials can be crafted using both “top-down” methods, involving the exfoliation of bulk materials, and “bottom-up” techniques, such as the formation of colloidal nanosheets and nanoparticles [8,9].

Understanding the fundamental thermophysical properties is crucial for modeling, developing, and optimizing deposition procedures for thin films, as well as for single crystal growth. Additionally, this knowledge plays a vital role in device development, as material characteristics like heat capacity and thermal conductivity are pivotal for creating high-performance devices tailored for industrial applications.

Although the ab initio techniques of electronic structure calculations may offer an alternative and feasible approach to assess these characteristics, as recently demonstrated on some layered structures and interfaces [10,11], as well as on Ga and In monochalcogenides [12], the experimental data obtained on real materials are indeed highly valuable, in particular for defected and disordered structures that must be theoretically modeled by ordered superstructures. This is also the case of Ga_2_Se_3_, Ga_2_Te_3_, and In_2_Te_3_, which crystallize in a disordered zinc blende structure with 1/3 of vacancies on a cation sublattice. This approach was thus applied in this study to calculate the enthalpy of formation of Ga_2_Te_3_, while the ordered counterparts were considered for Ga_2_Se_3_ and In_2_Te_3_.

## 2. Materials and Methods

### 2.1. Synthesis

Gallium, indium, sulfur, selenium, and tellurium were used in a form of granules (1–6 mm, 99.9999% purity, Wuhan XinRong New Materials Co., Wuhan, China). Gallium and indium chalcogenides of general formula M_2_Ch_3_ were produced through a direct reaction from elements in a quartz ampoule under a high vacuum. Quartz ampoules with dimensions of 25 × 120 mm and a wall thickness of 3 mm were used for the synthesis of gallium selenide, gallium telluride, and all indium chalcogenides. For the synthesis of Ga_2_S_3,_ an ampoule of dimensions 30 × 280 mm with a 3 mm wall thickness was used. For the synthesis, elements corresponding to 15 g of M_2_Ch_3_ with a precision better than ±1 mg were placed in the ampoule, which was further evacuated to the pressure of 1 × 10^−3^ Pa using a diffusion pump and sealed with an oxygen–hydrogen torch. The ampoules were heated at a heating rate of 5 °C/min to a temperature of about 50 °C above the melting point of the respective chalcogenide and kept at this temperature for 6 h. Subsequent cooling to room temperature was carried out at a rate of 0.2 °C/min. The maximum synthesis temperatures were 1050 °C for Ga_2_S_3_ and Ga_2_Se_3_, 850 °C for Ga_2_Te_3_, 950 °C for In_2_Se_3_, 1100 °C for In_2_S_3_, and 720 °C for In_2_Te_3_. A longer ampoule was used for the synthesis of Ga_2_S_3_ and the cold part of the ampoule was kept at 500 °C to avoid a high pressure of gaseous sulfur, since the reaction of elemental gallium and sulfur only occurs at temperatures exceeding 800 °C. The ampoules were opened in an argon atmosphere (glovebox) and the products were ground inside the glovebox using an agate mortar and pestle and sieved to a particle size of −100 mesh. In_2_Se_3_ with a layered structure in a bulk single-crystal form was used for the measurements.

### 2.2. Characterization

**Figure 1:**
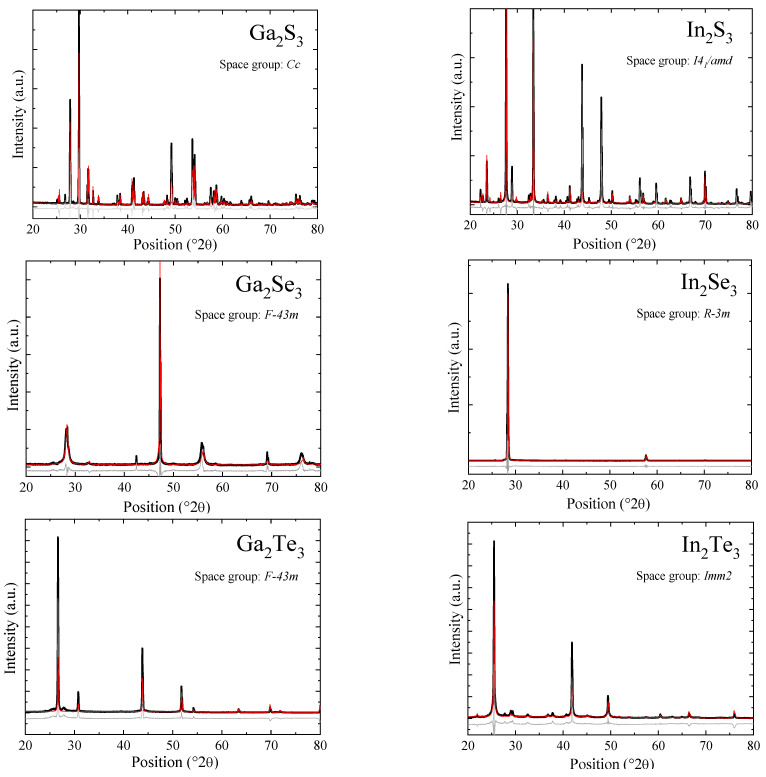
X-ray diffraction patterns of indium and gallium chalcogenides (black) and their Rietveld refinement (calculated profile—red, difference profile—gray).

The X-ray diffraction was performed on all six indium(III) and gallium(III) chalcogenides to identify the phase composition. X-ray diffraction patterns (Figure 1) show the single-phase composition of the synthesized chalcogenides.

Indium sulfide crystalized in a tetragonal structure (space group *I4_1_*/*amd*). Indium selenide reveals a rhombohedral layered structure (space group *R-3mH*), and the X-ray diffractogram exhibits a preferential orientation due to the layered structure. Gallium telluride and gallium selenide crystallize in a cubic structure (space group *F-43m*) derived from the zinc blende type with vacancies disordered on the cation sublattice, while gallium sulfide and indium telluride represent, respectively, ordered monoclinic (space group *Cc*) and orthorhombic (*Imm2*) forms of the metal-deficient zinc blende structure. The structure parameters obtained using Rietveld refinement (Topas program) based on the space groups given above are given in Table 1.

**Figure 2:**
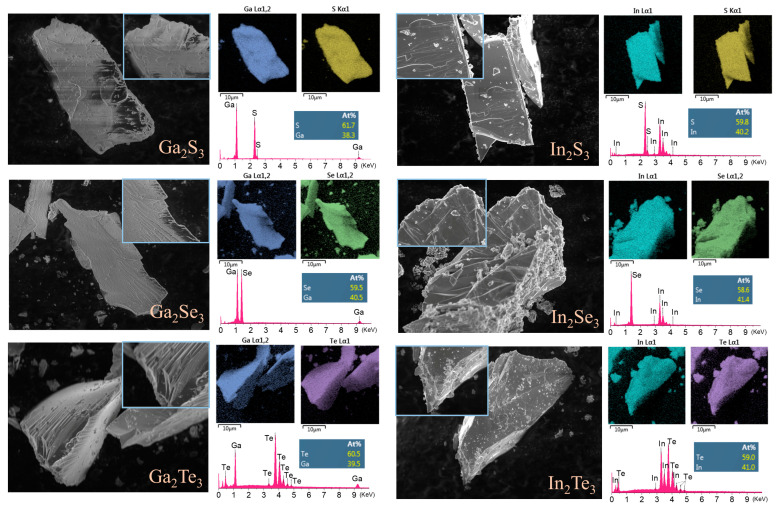
SEM images, elemental mappings, and EDS spectrum of prepared indium and gallium chalcogenides.

The morphologies and chemical compositions of the synthesized indium(III) and gallium(III) chalcogenides were meticulously characterized using scanning electron microscopy (SEM, Tescan MAIA 3, Dortmund, Germany) coupled with energy-dispersive X-ray spectroscopy (EDS, Oxford Instruments, Abingdon-on-Thames, UK), as shown in Figure 2.

The SEM analysis revealed a distinctive layered stacking morphology present in all chalcogenide compounds, encompassing both gallium and indium variants: Ga_2_S_3_, Ga_2_Se_3_, Ga_2_Te_3_, as well as In_2_S_3_, In_2_Se_3_, and In_2_Te_3_. Elemental mapping through EDS showcased a uniform distribution of constituent elements within the material matrix. Additionally, the observed elemental ratios closely aligned with the stoichiometric 2:3 ratio for gallium/indium to chalcogen, highlighting the precision of the synthetic approach. These findings affirm the successful formation of the target compounds, emphasizing their desired chemical composition and structural integrity.

**Figure 3:**
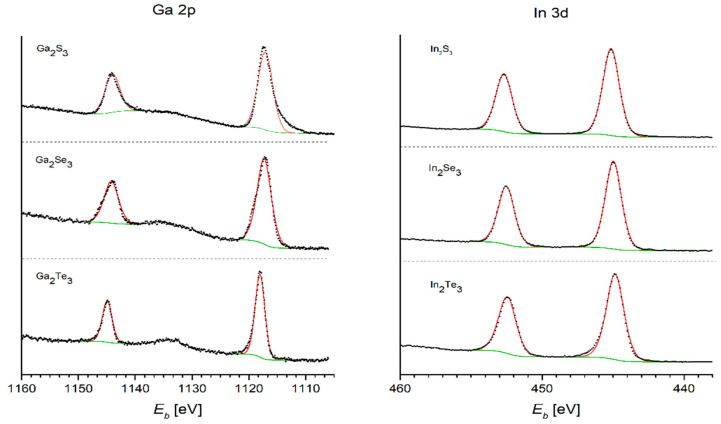
The Ga-2*p* (**left**) and In-3*d* (**right**) core regions of the XPS spectra of the chalcogenides of interest. Black dots represent the acquired data, the green curves the applied background correction, the red curves the fits.

The composition of all six materials was further confirmed with X-ray photoelectron spectroscopy (XPS) using a SPECS spectrometer equipped with a monochromatic Al Kα X-ray source (1486.7 eV) and a hemispherical electron analyzer, Phoibos 150. Figure 3 displays high-resolution spectra of Ga-2*p* and In-3*d* core regions of the materials. In the gallium-containing chalcogenides, two broad signals are evident—the Ga-2*p*_3/2_ and Ga-2*p*_1/2_ core levels are situated at approximately 1118 and 1145 eV, respectively, and could be fitted with a single component each. Similarly, the spectra of the indium-based chalcogenides exhibit two broad signals—the In-3*d*_5/2_ and In-3*d*_3/2_ core levels are located at approximately 453 and 445 eV, respectively. Like the gallium derivatives, these could be fitted with a single component each. The binding energy values correspond to the expected values for this class of compounds.

Similarly, the spectra of the core regions of the chalcogenides (S-2*p*, Se-3*d*, and Te-3*d*, respectively) show the characteristics expected for this type of compounds (Figures S1–S3). The S-2*p* regions of both sulfides show a single signal locate at ~162 eV, with well-resolved S-2*p_1/2_* and S-2*p_3/2_* components in the case of In_2_S_3_. Both selenides (In_2_Se_3_ as well as Ga_2_Se_3_) show one broad signal in the Se-3*d* region at ~54 eV that could be fitted with a single pair of the 3*d_3/2_* and 3*d_5/2_* components each. There are two well-resolved signals in the spectra of the Te-3*d* core region of both, In_2_Te_3_ as well as Ga_2_Te_3_, located at ~573 eV (3*d_5/2_*) and ~583 eV (3*d_3/2_*), respectively. Each of the signals could be fitted with a single component.

**Figure 4:**
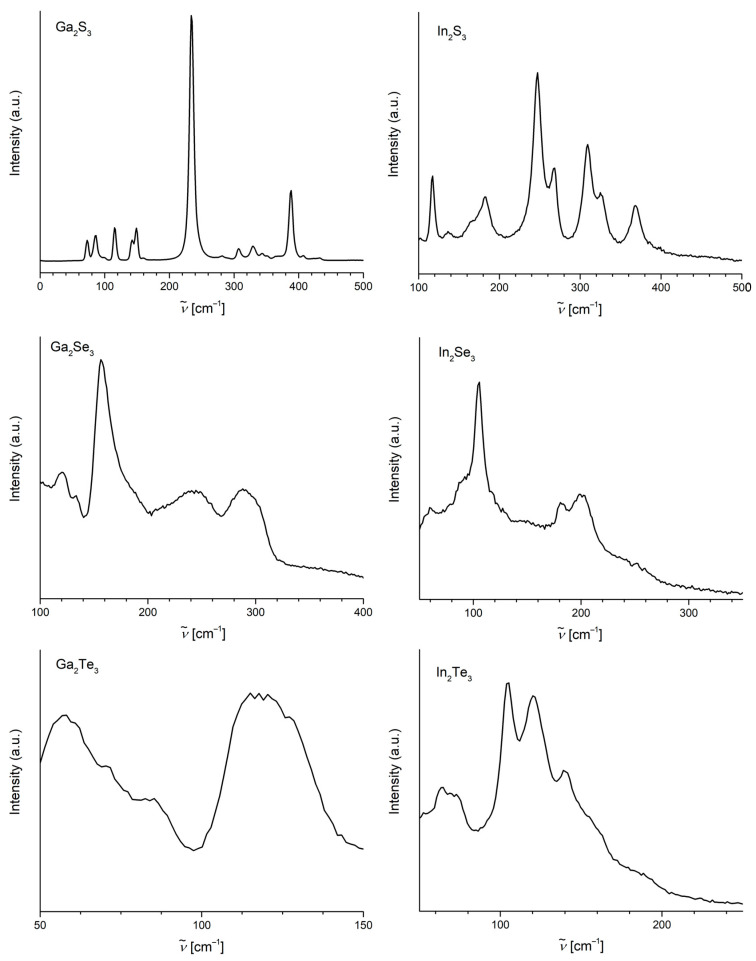
Raman spectra of the chalcogenides.

The Raman spectra of all the chalcogenides were recorded using a 532 nm Nd laser with low incident power and a 20× objective lens. There are two main resonance peaks in the spectrum of Ga_2_S_3_ (Figure 4) identified at 235 and 389 cm^−1^, assigned to the A_1_ and F_2_ modes, respectively [13], accompanied with several lower intensity peaks in the regions 70–170 and 300–360 cm^−1^. The spectra of the other two gallium(III) chalcogenides, Ga_2_Se_3_ and Ga_2_Te_3_, contain no sharp peaks but not-well-resolved broad signals with the maximum intensities at 157, 243, and 290 cm^−1^ for the selenide and 58 and 118 cm^−1^ for the telluride. The shape and quality of these spectra correspond to the data reported [14,15]. In the Raman spectrum of In2S3, there are several well-resolved peaks that could be assigned to the A_1g_ modes (245, 309, and 369 cm^−1^), E_g_ mode (269 cm^−1^), and F_2g_ modes (117, 183, and 326 cm^−1^) [16]. Similarly to the heavier gallium chalcogenides, the spectra of indium selenide and telluride are also less resolved than the spectrum of the sulfide (Figure 4). In the spectrum of In_2_Se_3_, there are three major signals at 106, 181, and 200 cm^−1^ assigned to the A_1_ modes [17]. The spectrum of In2Te3 corresponds to the only reported spectrum of this compound [18], with four main features at 63, 105, 121, and 140 cm^−1^.

### 2.3. Calorimetric Measurements

A Tian–Calvet-type calorimeter (SETARAM μDSC IIIa, Caluire, France) was used to determine the heat capacity in the temperature range 258–358 K. The heat capacities were obtained using a continuous method [19]. A three-step procedure was used, where the reference cell was always empty, while the measuring cell was empty, filled with a reference substance (synthetic sapphire, NIST standard reference material No. 720, Gaithersburg, MD, USA) and a sample. The combined expanded uncertainty of the heat capacity measurements (confidence level 0.95) is estimated to be *U*_c_(*C_p_*_m_) = 0.01 *C_p_*_m_ [20].

Physical Property Measurement System (PPMS) Model 6000 EverCool II (Quantum Design, San Diego, CA, USA), equipped with a heat capacity module (^4^He, *T*_min_ = 1.8 K), was used for the heat capacity measurements in the low-temperature region (2–300 K). The measurements were performed using the relaxation method under a high vacuum (pressure of 10^−2^ ± 10^−3^ Pa) to avoid heat loss through the exchange gas. The samples were wrapped in a copper foil and pressed into a pellet (with the exception of In_2_Se_3_, which was in the form of a monocrystal). These pellets were then mounted on a calorimeter platform using cryogenic grease, Apiezon N (supplied by the calorimeter manufacturer Quantum Design) [21]. The sample heat capacity was obtained as the difference between two data sets resulting from the sample run and the addenda (a blank sample holder with Apiezon N) performed under identical conditions. The uncertainty of heat capacity obtained using PPMS was recently investigated in our laboratory [21,22]. Based on tests with several compounds [21,22], the combined expanded uncertainty (0.95 level of confidence) of the heat capacity measurements is estimated to be 10 percent below 10 K, 3 percent in the temperature range of 10–40 K, and 2 percent in the temperature range of 40–300 K. To enhance the accuracy of the PPMS data, the results from this calorimeter were slightly adjusted to align with the results from the more accurate SETARAM μDSC IIIa, following common practice [23].

The following equation proposed by Archer [24] was used to describe temperature dependence of heat capacity in a wide temperature range (including literature heat capacities obtained using adiabatic calorimetry and data obtained using Quantum Design PPMS):

(1) $$C_{pm}^{o}/C_{pm}^{\mathrm{ref}}={\left(\frac{T}{T^{\mathrm{ref}}f(T)+bT}\right)}^{3}$$

where *T*^ref^ = 1 K and $C_{pm}^{\mathrm{ref}}$ = 1 J·K^−1^∙mol^−1^ and

(2) $$f(T)=a_{i}{\left(T-T_{i}\right)}^{3}+b_{i}{\left(T-T_{i}\right)}^{2}+c_{i}\left(T-T_{i}\right)+d_{i}$$

where only one parameter, *d_i_*, needs to be optimized for each temperature interval, while the values of the other three are determined using the continuity and smoothness constraints of the resulting temperature dependence. The parameter *b* is determined from the slope of *f*(*T*) at temperatures greater than 70 K [24].

### 2.4. DFT Calculations

The electronic structure calculations were performed within the VASP 5.4 program as implemented in the MedeA 3.6 software package using a Projector Augmented Waves method (PAW) [25] and a Generalized Gradient Approximation (GGA) with a PBE parametrization scheme [26]. The integration was performed using a tetrahedron method with Blöchl corrections on a mesh of *k*-points within the first Brillouin zone with a density of 0.25 Å^−1^. The structure parameters of group 13 sesqui-chalcogenides given in Table 1 were considered, with the exception of Ga_2_Se_3_ whose ordered form with monoclinic *Cc* symmetry (analogous to Ga_2_S_3_) was adopted. The obtained total energies were referred to the constituent elements in their ground state structure forms to evaluate the respective enthalpies of formation. 

## 3. Results

### 3.1. Heat Capacities

The experimental heat capacities obtained in this work using SETARAM μDSC IIIa and Quantum Design PPMS are listed in the Supplementary Materials (SMs) in Tables S1–S12. The available literature data on solid-phase heat capacities are summarized in Table 2. Selected experimental data from Table 2 (written in bold) were fitted to Equations (1) and (2), the parameters of which are listed in Table 3.

**Figure 5:**
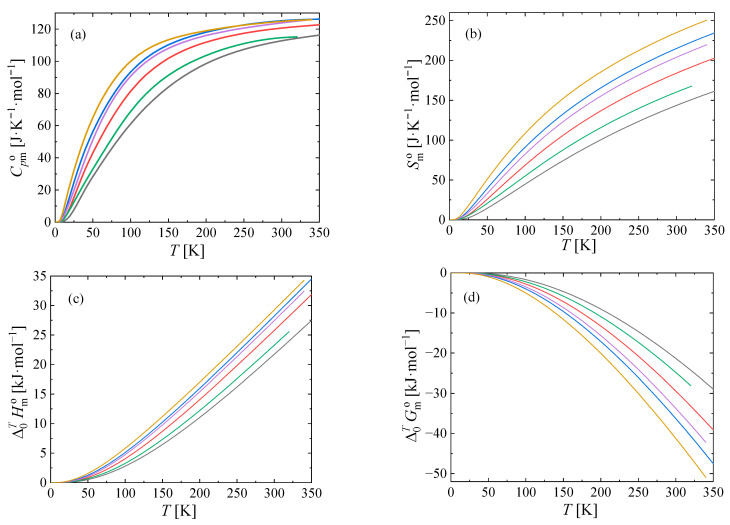
Standard molar thermodynamic functions at *p* = 0.1 MPa. Ga_2_S_3_ (black 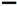), Ga_2_Se_3_ (red 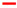), Ga_2_Te_3_ (blue 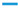), In_2_S_3_ (green 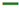), In_2_Se_3_ (purple 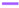), In_2_Te_3_ (olive 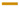). (**a**) Isobaric heat capacity, (**b**) entropy, (**c**) enthalpy, and (**d**) Gibbs energy.

The thermodynamic functions obtained using Equations (1) and (2) are tabulated at 298.15 K in Table 4, and at other temperatures in Tables S13–S18 in the SM, and shown in Figure 5.

**Figure 6:**
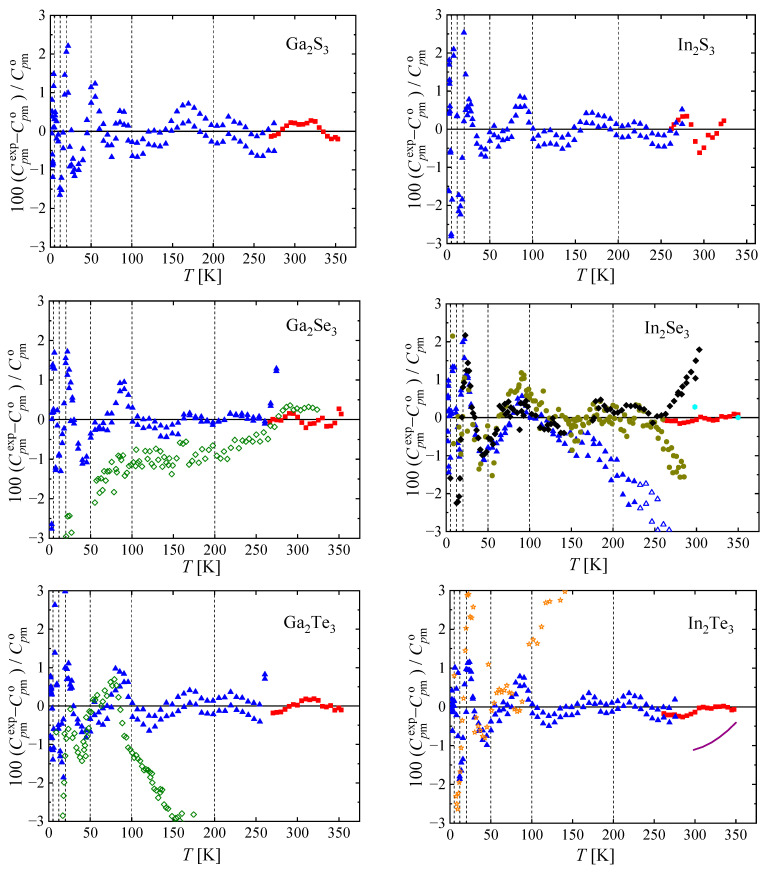
Relative deviations $100\left(C_{pm}^{\exp}-C_{pm}^{o}\right)/C_{pm}^{o}$ of individual experimental heat capacities $C_{pm}^{\exp}$ from values $C_{pm}^{o}$ calculated by means of Equations (1) and (2) with parameters from Table 2. Red 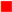, this work (Tian–Calvet calorimetry); blue 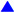, this work (relaxation calorimetry); purple 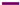, Knacke et al. [27] (for all compounds except In_2_Te_3_, all data by Knacke et al. [27] are out of scale); green 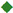, Tyurin et al. [28,29]; olive 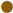, Boehnke et al. [31]; black 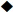, Koshchenko et al. [30]; orange 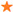, Zlomanov et al. [33]; cyan 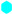, Mills [32]. Vertical lines mark knot temperatures *T_i_*. Data represented by filled symbols have been used to obtain parameters of Equations (1) and (2). Relaxation calorimetry data of this work for In_2_Se_3_ above 230 K have been excluded due to low coupling.

The experimental heat capacities for all studied chalcogenides are compared with the smoothed values obtained using Equations (1) and (2) in Figure 6. The deviations of the selected experimental data from the smoothed values generally do not exceed 2% (except for a few outliers, which are not considered in the correlation).

### 3.2. Enthalpies of Formation

The total energies calculated in VASP with the PAW-GGA method were first recalculated to enthalpies of formation at *T* = 0 K using the total energies of constituent elements in their stable forms obtained with the same technique. As mentioned, the refined lattice parameters and atomic positions were used for the sesqui-chalcogenides (see Table 1, except for Ga_2_Se_3_, see Section 2.4), while the structure data of elements were adopted from the InfoMatica-ICSD database implemented in the MedeA software. The disordered structure of Ga_2_Se_3_ of the zinc blende type was modeled in terms of a 1 × 1 × 3 supercell (12 formula units) with four vacant Ga positions (000, ½ ½ ⅓, ½ 0 ½, ½ ½ ⅔). The enthalpies of formation at 0 K were further recalculated to cohesive energies using the enthalpies of vaporization of the constituent elements [34] and to the enthalpies of formation at a reference temperature of *T* = 298.15 K (see Table 4) using the relative enthalpies 0–>298 also given in Table 4 and the analogous relative enthalpies of elements [35]. 

## 4. Discussion

The heat capacity of the studied chalcogenides was measured using the SETARAM µDSC IIIa and QuantumDesign PPMS calorimeters. In_2_Se_3_ was measured in the form of a crystal, and the rest of the chalcogenides were grinded into powders and pressed into pellets covered with copper foil.

The results were compared with available literature (see Figure 6). A handbook by Knacke et al. [27] provides parameters for the heat capacity of these chalcogenides in a wide temperature range. These values differ significantly from our measurements (by 4 to 20%) with the exception of In_2_Te_3,_ which agreed with our measurements within 1% in the range of overlap—from 300 to 350 K. The adiabatic heat capacity data by Tyurin et al. [28] agree with our measurements within 2% above 50 K in the case of Ga_2_Se_3._ Below 50 K, the difference increases to 20% at 15 K, the lowest point of Tyurin et al. [28]. Therefore, we decided not to include this data set in our correlation. On the other hand, adiabatic data by Tyurin et al. [29], Koschenko et al. [30], Boenke et al. [31], and Zlomanov et al. [33] are in good agreement with our measurements, with the exception of the tellurides (Ga_2_Te_3_, In_2_Te_3_), which deviate for temperatures above 100 K (with maximum deviation of 5% for In_2_Te_3_ [33] and 9% for Ga_2_Te_3_ [29]). However, at temperatures lower than 100 K, the agreement is also very good. The reason for the discrepancy observed above 100 K remains unclear and requires further elucidation.

**Figure 7:**
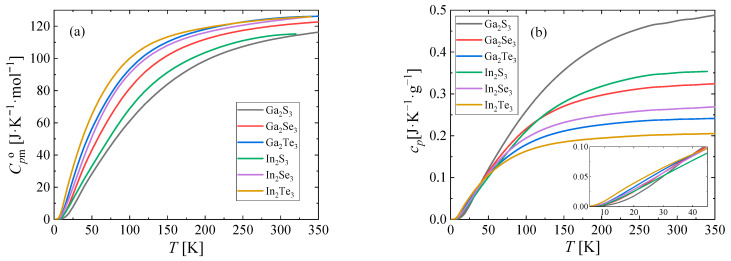
Heat capacity of Ga and In sesqui-chalcogenides studied in this work. (**a**) Molar, (**b**) specific.

The resulting heat capacities were fitted with the reverse spline function suggested by Archer et al. [24] and their comparison is shown in Figure 7.

As we can see in Figure 7a, the order of heat capacities (from highest to lowest) is In_2_ > Ga_2_ and Te_3_ > Se_3_ > S_3_. This clear trend is a manifestation of (i) increasing molar masses (*M*(Ga_2_S_3_) = 235.64; *M*(In_2_Te_3_) = 612.44) and (ii) softer force constants related to chemical bond weakening with increasing atomic size (due to less effective valence orbital overlap), both resulting in an enhanced phonon mode population at lower temperatures. In contrast, if we compare their specific heat capacities instead (Figure 7b), we obtain an exact opposite order at ambient temperatures. Interestingly, the specific heat capacities cross over at about 40 K and at very low temperatures, the order of specific heat capacities being the same as for molar heat capacities (see the inset of Figure 7b).

**Figure 8:**
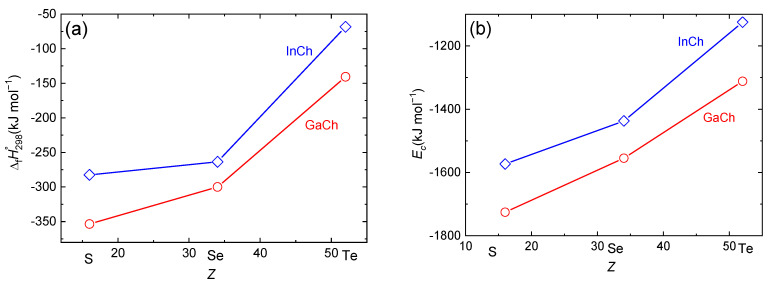
Enthalpies of formation (**a**) and cohesive energies (**b**) of Ga and In sesqui-chalcogenides evaluated from DFT calculations.

The calculated enthalpies of formation at 298 K and cohesive energies at 0 K referred, respectively, to elements in their stable solid forms and to a noninteracting monoatomic gas are plotted in Figure 8a,b against the atomic number of chalcogen. In both cases, a decreasing stability with an increasing atomic number of group 16 as well as group 13 elements is apparently a result of decreasing strength of the covalent bond due to a less effective overlap of more diffused valence orbitals, which is also in line with the observed trend in molar heat capacities.

Although the calculated enthalpies of formation reveal an expected trend, their values are underestimated with respect to the data given in tables published by Knacke et al. [27]. However, the heat capacities of this work also do not agree well with Knacke et al. [27] so the two discrepancies might compensate each other in cases where the enthalpies of formation were assessed from high-temperature equilibrium data.

## 5. Conclusions

In this work, six sesqui-chalcogenides of indium and gallium were synthesized and characterized using XRD, SEM, XPS, and Raman spectroscopy. The heat capacity of gallium and indium sesqui-chalcogenides was determined by means of relaxation calorimetry (QuantumDesign PPMS) and Tian–Calvet calorimetry (SETARAM µDSC IIIa). Both techniques yielded consistent results comparable to available literature data obtained using adiabatic calorimetry. Moreover, the enthalpies of formation were assessed from DFT calculation of total energies. Experimental and theoretical data of this work, along with selected literature data, were used to derive standard thermodynamic data (enthalpy, entropy, Gibbs energy) in the temperature range from 0 K to 340 K.

## Figures and Tables

**Table 1:** Lattice parameters of Ga_2_S_3_, Ga_2_Se_3_, Ga_2_Te_3_, In_2_S_3_, In_2_Se_3_, and In_2_Te_3_ as obtained with Rietveld refinement of the recorded X-ray diffraction patterns using Topas program.

	Ga_2_S_3_	Ga_2_Se_3_	Ga_2_Te_3_	In_2_S_3_	In_2_Se_3_	In_2_Te_3_
Space group	*Cc*	*F-43m*	*F-43m*	*I4_1_/amd*	*R-3mH*	*Imm2*
Lattice parameters/Å	a = 11.1172 b = °6.4041 c = °7.0305 ß = °121.19	a = 5.4235	a = 5.903	a = 7.617 c = 32.32	a = °3.978 c = 28.99	a = 13.083 b = °4.361 c = °6.168

**Table 2:** Overview of the Literature Crystal Heat Capacities of Ga_2_S_3_, Ga_2_Se_3_, Ga_2_Te_3_, In_2_S_3_, In_2_Se_3_, and In_2_Te_3_.

Reference	*N* ^a^	(*T*_min_ − *T*_max_)/K	*u_r_*(*C_p_*_m_)/% ^b^	Method
Ga_2_S_3_				
Knacke et al. [27]	S ^c^	298–1213	nosp.	nosp.
**This work**	**18**	**271–353**	**1.0**	**Tian–Calvet**
**This work**	**126**	**2–302**	**^d^**	**Relaxation**
Ga_2_Se_3_				
Knacke et al. [27]	S ^c^	298–1278	nosp.	nosp.
Tyurin et al. [28]	101	15–324	0.2	Adiabatic
**This work**	**19**	**266–353**	**1.0**	**Tian–Calvet**
**This work**	**125**	**2–302**	**^d^**	**Relaxation**
Ga_2_Te_3_				
Knacke et al. [27]	S ^c^	298–1063	nosp.	nosp.
Tyurin et al. [29]	134	9–310	0.2	Adiabatic
**This work**	**19**	**271–353**	**1.0**	**Tian–Calvet**
**This work**	**132**	**2–303**	**^d^**	**Relaxation**
In_2_S_3_				
Knacke et al. [27]	S ^c^	298–660	nosp.	nosp.
**This work**	**14**	**262–323**	**1.0**	**Tian–Calvet**
**This work**	**125**	**2–302**	**^d^**	**Relaxation**
In_2_Se_3_				
Knacke et al. [27]	S ^c^	298–470	nosp.	nosp.
**Koshchenko et al.** [30]	**99**	**4–304**	**0.5**	**Adiabatic**
**Boehnke et al.** [31]	**138**	**6–285**	**0.1**	**Adiabatic**
**Mills** [32]	**5**	**298–486**	**0.5**	**DSC**
**This work**	**19**	**262–350**	**1.0**	**Tian–Calvet**
**This work**	**104**	**2–226**	**^d^**	**Relaxation**
This work	22	232–302	^d^	Relaxation
In_2_Te_3_				
Knacke et al. [27]	S ^c^	298–898	nosp.	nosp.
Zlomanov et al. [33]	75	5–313	0.2	Adiabatic
**This work**	**19**	**262–348**	**1.0**	**Tian–Calvet**
**This work**	**126**	**2–303**	**^d^**	**Relaxation**

^a^ *N* = number of data points. ^b^ *u*_r_(*C_p_*_m_) stands for relative uncertainty in heat capacity as stated by the authors. ^c^ S stands for smoothed data (given in the form of an equation); ^d^ the combined expanded uncertainty of heat capacity with 0.95 level of confidence (*k* = 2) of PPMS using thermal relaxation measurement technique is *U*_c_(*C_p_*_m_) = 0.1 *C_p_*_m_ below 10 K; *U*_c_(*C_p_*_m_) = 0.03 *C_p_*_m_ in temperature range 10 to 40 K; *U*_c_(*C_p_*_m_) = 0.02 *C_p_*_m_ in temperature range 40 to 300 K.

**Table 3:** Parameters of Equations (1) and (2) for Crystal Heat Capacities in J·K^−1^ mol^−1^.

*a*_i_	*b*_i_	*c*_i_	*d*_i_	*T*_i_/K	*T*_i, max_/K	*N* ^a^	*s*_r_ ^b^
		Ga_2_S_3_		*b* = 0.18			
1.10116 × 10^−2^	−2.03631 × 10^−1^	9.61860 × 10^−1^	1.23920 × 10^1^	0	5	14	0.93
2.81063 × 10^−3^	−3.84571 × 10^−2^	−2.48579 × 10^−1^	1.34870 × 10^1^	5	12	8	1.17
−6.13516 × 10^−4^	2.05661 × 10^−2^	−3.73816 × 10^−1^	1.08266 × 10^1^	12	20	8	2.74
−6.72147 × 10^−5^	5.84174 × 10^−3^	−1.62553 × 10^−1^	8.83815	20	50	17	1.09
1.43753 × 10^−6^	−2.07582 × 10^−4^	6.47156 × 10^−3^	7.40433	50	100	19	0.56
1.74864 × 10^−7^	8.04748 × 10^−6^	−3.50518 × 10^−3^	7.38864	100	200	30	0.40
−1.34810 × 10^−7^	6.05065 × 10^−5^	3.35023 × 10^−3^	7.29346	200	353	48	0.36
		Ga_2_Se_3_		*b* = 0.19			
5.69538 × 10^−2^	−8.73244 × 10^−1^	4.22755	2.28158	0	5	10	1.60
1.75173 × 10^−3^	−1.89368 × 10^−2^	−2.33356 × 10^−1^	8.70745	5	12	10	1.00
−6.36373 × 10^−4^	1.78496 × 10^−2^	−2.40966 × 10^−1^	6.74691	12	20	8	1.07
−3.14760 × 10^−5^	2.57666 × 10^−3^	−7.75553 × 10^−2^	5.63574	20	50	16	0.77
2.57153 × 10^−6^	−2.56181 × 10^−4^	−7.94088 × 10^−3^	4.77822	50	100	19	0.42
−3.97876 × 10^−7^	1.29549 × 10^−4^	−1.42725 × 10^−2^	4.06217	100	200	30	0.26
7.82412 × 10^−8^	1.01860 × 10^−5^	−2.99051 × 10^−4^	3.53252	200	353	37	0.18
		Ga_2_Te_3_		*b* = 0.19			
1.11589 × 10^−2^	−1.47396 × 10^−1^	2.08914 × 10^−1^	7.96519	0	5	14	0.90
−1.70943 × 10^−4^	1.99869 × 10^−2^	−4.28133 × 10^−1^	6.71971	5	12	8	1.70
−6.39490 × 10^−4^	1.63972 × 10^−2^	−1.73444 × 10^−1^	4.64351	12	20	8	2.18
−1.32927 × 10^−5^	1.04939 × 10^−3^	−3.38717 × 10^−2^	3.97796	20	50	16	0.74
1.62523 × 10^−6^	−1.46950 × 10^−4^	−6.79852 × 10^−3^	3.54735	50	100	20	0.56
−3.13065 × 10^−7^	9.68342 × 10^−5^	−9.30432 × 10^−3^	3.04321	100	200	30	0.29
1.02416 × 10^−7^	2.91463 × 10^−6^	6.70564 × 10^−4^	2.76805	200	353	34	0.18
		In_2_S_3_		*b* = 0.19			
2.79528 × 10^−2^	−3.99655 × 10^−1^	1.29063	1.00431 × 10^1^	0	5	14	1.78
4.94311 × 10^−4^	1.96370 × 10^−2^	−6.09463 × 10^−1^	9.99897	5	12	8	3.11
−1.21050 × 10^−3^	3.00175 × 10^−2^	−2.61881 × 10^−1^	6.86449	12	20	8	2.61
−1.61253 × 10^−5^	9.65519 × 10^−4^	−1.40172 × 10^−2^	6.07079	20	50	17	0.69
4.10646 × 10^−6^	−4.85758 × 10^−4^	3.75668 × 10^−4^	6.08385	50	100	19	0.48
−3.81795 × 10^−7^	1.30212 × 10^−4^	−1.74016 × 10^−2^	5.40155	100	200	30	0.29
2.88444 × 10^−7^	1.56732 × 10^−5^	−2.81313 × 10^−3^	4.58171	200	323	38	0.33
		In_2_Se_3_		*b* = 0.18			
1.51705 × 10^−2^	−1.95861 × 10^−1^	3.7698 × 108^−1^	8.19467	0	5	14	0.94
−8.54900 × 10^−4^	3.16966 × 10^−2^	−4.43834 × 10^−1^	7.07940	5	12	14	3.24
−5.67811 × 10^−4^	1.37437 × 10^−2^	−1.25752 × 10^−1^	5.23246	12	20	18	1.71
−3.28446 × 10^−7^	1.16225 × 10^−4^	−1.48729 × 10^−2^	4.81532	20	50	41	1.03
9.00517 × 10^−8^	8.66649 × 10^−5^	−8.78617 × 10^−3^	4.46487	50	100	66	0.59
−3.04634 × 10^−7^	1.00173 × 10^−4^	5.55706 × 10^−4^	4.25348	100	200	100	0.31
−5.99553 × 10^−8^	8.78254 × 10^−6^	1.14512 × 10^−2^	5.00614	200	350	56	0.20
		In_2_Te_3_		*b* = 0.19			
1.46034 × 10^−2^	−1.76452 × 10^−1^	1.85958 × 10^−1^	6.88854	0	5	14	0.33
−1.45817 × 10^−3^	4.25984 × 10^−2^	−4.83311 × 10^−1^	5.23244	5	12	8	1.60
−5.00450 × 10^−4^	1.19768 × 10^−2^	−1.01284 × 10^−1^	3.43644	12	20	8	2.36
−9.09745 × 10^−8^	−3.39669 × 10^−5^	−5.74150 × 10^−3^	3.13645	20	50	16	0.87
9.49425 × 10^−7^	−4.21546 × 10^−5^	−8.02514 × 10^−3^	2.93118	50	100	20	0.45
−3.69997 × 10^−7^	1.00259 × 10^−4^	−5.11991 × 10^−3^	2.54321	100	200	30	0.23
9.21853 × 10^−8^	−1.07400 × 10^−5^	3.83202 × 10^−3^	2.66382	200	348	49	0.28

^a^ *N* stands for number of experimental data points in given temperature interval used for correlation. ^b^ $s_{r}=100{\left\{\sum_{i=1}^{n}{\left[\left(C_{pm}^{\exp}-C_{pm}^{o}\right)/C_{pm}^{o}\right]}_{i}^{2}/\left(N-m\right)\right\}}^{1/2}$, where $C_{pm}^{\exp}$ and $C_{pm}^{o}$ are the experimental and calculated (Equations (1) and (2)) heat capacity, *N* is the number of fitted data points, and *m* is the number of independent adjustable parameters.

**Table 4:** Standard Thermodynamic Functions of Ga(III) and In(III) chalcogenides derived from heat capacity measurements and the calculated enthalpies of formation at *T* = 298.15 K and *p* = 0.1 MPa ^a^.

	$C_{pm}^{o}$/J·K^−1^·mol^−1^	∆*_f_H^°^*/kJ·mol^−1^	$S_{m}^{o}$/J·K^−1^·mol^−1^	$\Delta_{0}^{T}H_{m}^{o}$/kJ·mol^−1^	$\Delta_{0}^{T}G_{m}^{o}$/kJ·mol^−1^
Ga_2_S_3_	112.6	−353.5	142.8	21.51	−21.06
Ga_2_Se_3_	120.6	−299.9	183.2	25.54	−29.09
Ga_2_Te_3_	125.1	−140.7	214.2	27.97	−35.88
In_2_S_3_	114.6	−282.5	159.6	23.05	−24.53
In_2_Se_3_	123.8	−263.6	203.1	27.21	−33.36
In_2_Te_3_	124.7	−68.4	234.1	29.02	−40.78

^a^ The combined expanded uncertainty of heat capacity *U*_c_(*C_p_*_m_) as well as of all calculated thermodynamic values (with 0.95 level of confidence, *k* = 2) is *U*_c_(*X*) = 0.01 *X* in temperature range 260 to 340 K, where *X* represents the heat capacity or the thermodynamic property. Values are reported with one digit more than is justified by the experimental uncertainty to avoid round-off errors in calculations based on these results.

## Data Availability

The data presented in this study are available in the Supplementary Materials.

## Supplementary Materials

The following Supporting Information can be downloaded at: https://www.mdpi.com/article/10.3390/ma17020361/s1, X-ray photoelectron spectroscopy (XPS) results for the core regions of the chalcogenides (Figures S1–S3), Auxiliary properties describing the quality of PPMS measurement (Figures S4 and S5), Experimental heat capacity data obtained in this work (Tables S1–S12), Tabulated thermodynamic functions of studied sesqui-chalcogenides (Tables S13–S18).
