# Supplementary material for: Heat Capacity of Indium or Gallium Sesqui-Chalcogenides
Source: Materials (Basel). 2024 Jan 11;17(2):361. doi: 10.3390/ma17020361 (PMC10817357; doi:10.3390/ma17020361)
Supplement: Supplementary file 1 [file materials-17-00361-s001.zip › materials-2770206-supplementary.pdf]

Supplementary materials

Heat capacity of indium or gallium sesqui-chalcogenides

Květoslav Růžička [1], Václav Pokorný [1,2], Jan Plutnar [3], Iva Plutnarová [3], Bing Wu [3], Zdeněk Sofer [3] and David Sedmidubský [3,*]

1 Department of Physical Chemistry, Faculty of Chemical Engineering, University of Chemistry and Technology, Prague, Technická 5, 166 28 Prague, Czech Republic; ruzickak@vscht.cz (K.R.); pokorny@imc.cas.cz (V.P.)
2 Institute of Macromolecular Chemistry, Czech Academy of Sciences, Heyrovského Nám. 2, 162 06 Prague, Czech Republic
3 Department of Inorganic Chemistry, Faculty of Chemical Technology, University of Chemistry and Technology, Prague, Technická 5, 166 28 Prague, Czech Republic; jan.plutnar@vscht.cz (J.P.); iva.plutnarova@vscht.cz (I.P.); bing1.wu@vscht.cz (B.W.); zdenek.sofer@vscht.cz (Z.S.)
* Correspondence: sedmidub@vscht.cz

1. X-ray photoelectron spectroscopy (XPS) results for the core regions of the chalcogenides (S-2p, Se-3d, and Te-3d, respectively).

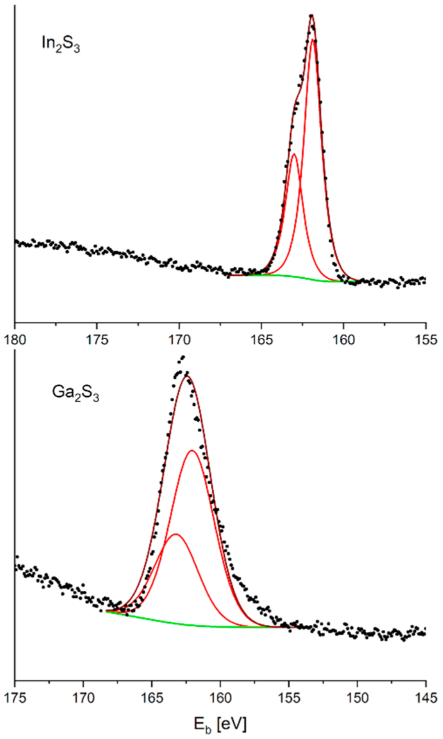

Figure S1. XPS spectrum of the S-2*p* core region of In_2S_3 (top) and Ga_2S_3 (bottom). Black dots represent the acquired data, the green lines represent the baseline fit, red lines represent the fitted components, the brown lines represent the sum of the fits.

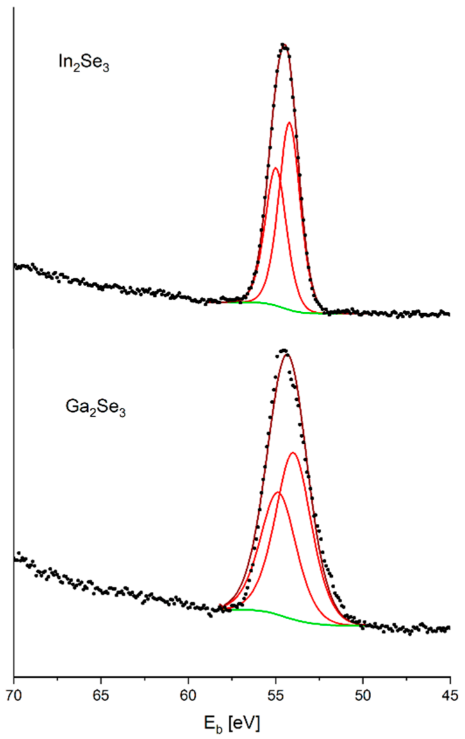

Figure S2. XPS spectrum of the Se-3*d* core region of In_2Se_3 (top) and Ga_2Se_3 (bottom). Black dots represent the acquired data, the green lines represent the baseline fit, red lines represent the fitted components, the brown lines represent the sum of the fits.

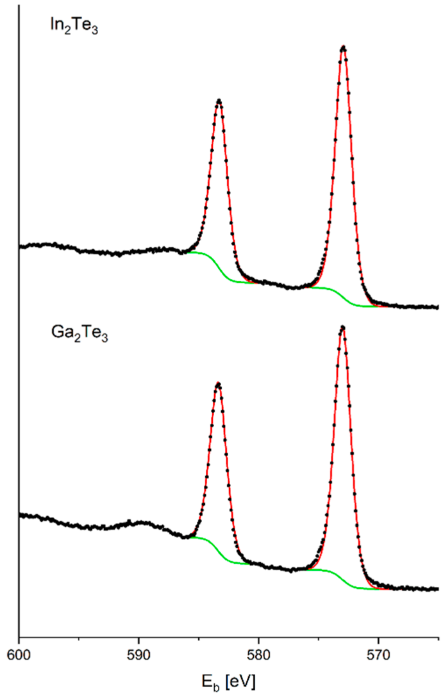

Figure S3. XPS spectrum of the Te-3*d* core region of In_2Te_3 (top) and Ga_2Te_3 (bottom). Black dots represent the acquired data, the green lines represent the baseline fit, and the red lines represent the fitted components.

2. Auxiliary properties describing the quality of PPMS measurement

Figure S4 shows the sample coupling during PPMS measurements. Sample coupling is a property describing the thermal contact between the sample and the platform. A lower value generally means higher uncertainty of the data. For reliable data, the coupling should be above 90%. This was not the case for In_2Se_3 above 270 K, and those points were therefore not considered in the correlation.

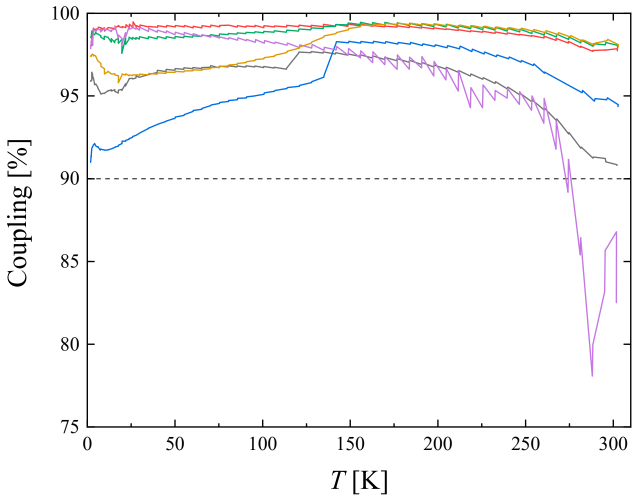

Figure S4. Sample coupling of PPMS samples. Ga_2S_3 (black —), Ga_2Se_3 (red —), Ga_2Te_3 (blue —), In_2S_3 (green —), In_2Se_3 (purple —), In_2Te_3 (olive —).

Figure S5 shows the relative contribution to heat capacity of different parts of the PPMS experimental setup. The lower contribution of the sample also increases uncertainty, and the sample should ideally contribute with at least 50% of the total heat capacity. That is, however, not always possible, especially with samples in copper foil. Bigger samples also usually have lower sample coupling, so a balance between these two factors has to be made.

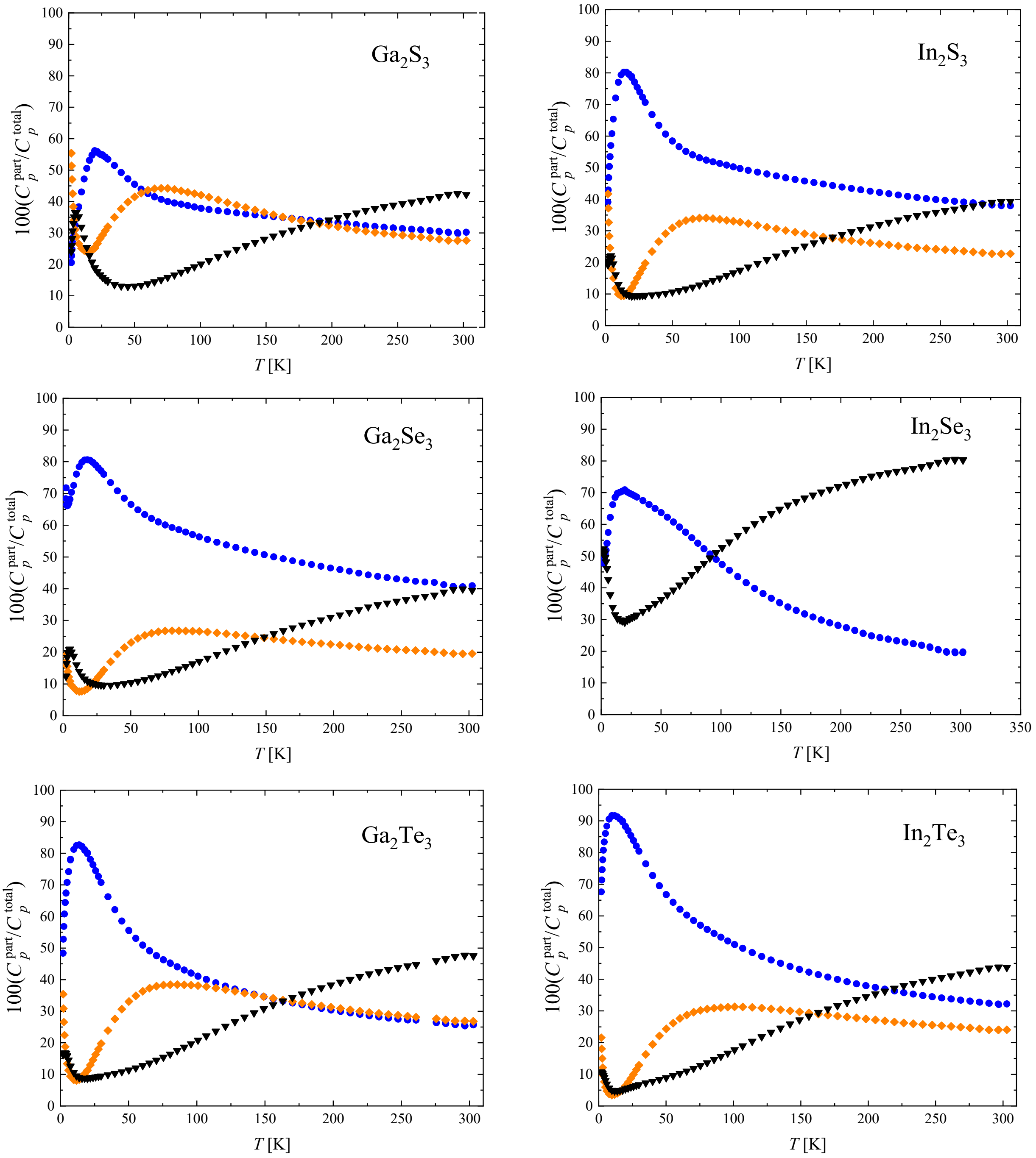

Figure S5. Relative contribution to heat capacity of different parts of the PPMS experimental setup: blue ●, sample; orange ◆, copper; black▼, addenda.

3. Experimental heat capacities

This section contains experimental heat capacity data obtained in this work by the means of Tian–Calvet calorimetry (apparatus SETARAM μDSC IIIa), and relaxation calorimetry (apparatus Quantum Design PPMS). Due to their lower accuracy, data obtained using the Quantum Design PPMS were slightly adjusted to agree with results from the more accurate SETARAM μDSC IIIa, as described in section 2.3 of the main manuscript. These correction factors are shown in the footnote of each table.

Table S1. Experimental heat capacity of Ga_2S_3 (in J K^{-1} mol^{-1}) obtained using Tian–Calvet calorimetry (SETARAM μDSC IIIa)[a].

T / K	C_{pm} /J·K^{-1}·mol^{-1}	δ_{rel}[b]
271.06	109.81	-0.13
275.00	110.26	-0.11
280.00	110.82	-0.07
285.00	111.46	0.06
290.00	112.02	0.14
295.00	112.57	0.22
300.00	112.99	0.21
305.00	113.37	0.18
310.00	113.77	0.18
315.00	114.19	0.21
320.00	114.64	0.28
325.00	114.97	0.25
330.00	115.13	0.10
335.00	115.35	-0.01
340.00	115.53	-0.13
345.00	115.77	-0.20
350.00	116.12	-0.16
352.83	116.24	-0.20

[a] Standard uncertainty u is $u(T)$ = 0.05 K, and the combined expanded uncertainty of the heat capacity is $U_c(C_{p,m}) = 0.01C_{p,m}$ (0.95 level of confidence). Values are reported with one digit more than is justified by the experimental uncertainty to avoid round-off errors in calculations based on these results.

[b] $\delta_{rel} = 100\times\left(C_{pm} - C^{o}_{pm}\right) / C^{o}_{pm}$, where heat capacity is calculated by means of Eqs. 1 and 2 with parameters from Table 2 in the main article.

Table S2. Experimental heat capacity of Ga_2S_3 (in J K^{-1} mol^{-1}) obtained using the relaxation technique (Quantum Design PPMS)[a].

T / K	C_{pm} / J·K⁻¹·mol⁻¹	δ_{rel} [b]	*T*/ K	C_{pm} / J·K⁻¹·mol⁻¹	δ_{rel} [b]
	Run 1			Run 2	
302.163	113.17	0.21	302.150	113.34	0.37
295.736	111.10	-1.14	295.453	112.79	0.38
288.727	111.84	0.09	288.456	112.60	0.79
281.782	110.80	-0.25	281.484	111.61	0.51
274.789	109.79	-0.51	274.503	110.55	0.20
267.795	109.04	-0.50	267.523	109.61	0.04
260.850	108.08	-0.64	260.561	108.69	-0.05
253.858	107.22	-0.64	253.571	107.72	-0.13
246.854	106.36	-0.56	246.578	106.84	-0.08
239.844	105.53	-0.40	239.575	106.02	0.10
232.856	104.59	-0.27	232.578	105.03	0.20
225.806	103.50	-0.19	225.554	103.91	0.24
218.791	102.37	-0.08	218.545	102.78	0.38
211.793	100.82	-0.28	211.557	101.20	0.15
204.790	99.328	-0.32	204.558	99.732	0.14
197.777	97.814	-0.27	197.558	98.183	0.16
190.780	96.283	-0.12	190.568	96.659	0.33
183.777	94.572	-0.02	183.570	94.960	0.45
176.754	92.760	0.12	176.545	93.149	0.60
169.705	90.792	0.25	169.501	91.148	0.71
162.728	88.539	0.20	162.530	88.887	0.67
155.675	86.067	0.09	155.469	86.356	0.51
148.663	83.477	0.01	148.454	83.611	0.26
141.729	80.562	-0.32	141.502	80.771	0.05
134.673	77.653	-0.39	134.454	77.839	-0.04
127.674	74.664	-0.37	127.463	74.857	0.01
120.689	71.496	-0.35	120.485	71.637	-0.01
113.714	67.957	-0.59	113.519	68.120	-0.21
106.681	64.308	-0.67	106.494	64.446	-0.30
100.680	61.089	-0.64	100.489	61.206	-0.28
95.603	58.463	-0.24	95.431	58.590	0.14
90.570	55.734	0.17	90.406	55.821	0.50
85.480	52.656	0.23	85.317	52.709	0.52
80.414	49.246	-0.20	80.254	49.337	0.19
75.331	45.733	-0.67	75.176	45.773	-0.36
70.313	42.532	-0.37	70.164	42.550	-0.09
65.222	39.102	-0.25	65.077	39.172	0.18
60.196	35.696	0.06	60.058	35.759	0.51
55.104	32.317	0.89	54.969	32.328	1.23
50.080	28.583	0.73	49.950	28.602	1.14
45.013	24.585	-0.05	44.888	24.574	0.29
39.941	20.420	-0.75	39.821	20.382	-0.46
34.744	15.972	-1.01	34.732	15.986	-0.85
29.676	11.509	-1.16	29.668	11.522	-0.99
27.799	9.9070	-1.06	27.749	9.8988	-0.71
25.761	8.3091	0.02	25.722	8.2033	-0.87

T / K	C_{pm} / J·K^{-1}·mol^{-1}	δ_{rel} [b]	T/ K	C_{pm} / J·K^{-1}·mol^{-1}	δ_{rel} [b]
	Run 1			Run 2	
23.872	6.7903	-0.41	23.817	6.7144	-0.91
21.756	5.4064	2.21	21.783	5.3608	0.99
19.670	4.1317	4.34	19.741	4.0842	2.06
17.634	2.8769	0.95	17.719	2.9346	1.46
15.689	1.9667	-0.04	15.730	1.9754	-0.44
13.683	1.2309	-1.21	13.700	1.2322	-1.52
11.717	0.714331	-1.47	11.720	0.7137	-1.65
9.674	0.366560	-0.09	9.690	0.36805	-0.27
7.607	0.160945	0.26	7.626	0.16157	-0.19
5.929	0.0717415	0.49	5.931	0.071758	0.41
4.932	0.0403732	0.31	4.932	0.040288	0.10
4.104	0.0234448	1.48	4.106	0.023406	1.17
3.433	0.013555	-0.84	3.430	0.013647	0.09
2.947	0.0087378	-0.79	2.948	0.0087107	-1.19
2.515	0.0055798	-0.61	2.512	0.0055455	-0.88
2.175	0.0037482	0.51	2.173	0.0037337	0.38
1.912	0.0026194	0.81	1.911	0.0025929	-0.06

[a] Standard uncertainty of temperature is $u(T)$=0.004 K, and the combined expanded uncertainty of heat capacity $U_c(C_{pm})$ with 0.95 level of confidence (k=2) is $U_c(C_{pm})$=0.1 C_{pm} below 10 K; $U_c(C_{pm})$=0.03 C_{pm} in temperature range 10 to 40 K; $U_c(C_{pm})$=0.02 C_{pm} in temperature range 40 to 300 K. Values are reported with more digits than is justified by the experimental uncertainty to avoid round-off errors in calculations based on these results. Measurements are performed in vacuum (residual pressure p<10^{-4} Pa). Excluded points are printed in gray. Experimental results from Quantum Design PPMS have been multiplied by a factor of 1.015 to agree with the more accurate SETARAM μDSC IIIa.

[b] $\delta_{rel} = 100\times\left(C_{pm} - C^{o}_{pm}\right) / C^{o}_{pm}$, where heat capacity is calculated by means of Eqs. 1 and 2 with parameters from Table 2 in the main article.

Table S3. Experimental heat capacity of Ga_2Se_3 (in J K^{-1} mol^{-1}) obtained using Tian–Calvet calorimetry (SETARAM μDSC IIIa)[a].

T / K	C_{pm} /J·K^{-1}·mol^{-1}	δ_{rel}[b]
266.36	118.58	-0.07
270.00	118.92	0.01
275.00	119.22	-0.01
280.00	119.52	-0.02
285.00	119.94	0.08
290.00	120.31	0.15
295.00	120.58	0.15
300.00	120.73	0.06
305.00	120.80	-0.08
310.00	120.88	-0.21
315.00	121.22	-0.11
320.00	121.44	-0.10
325.00	121.74	-0.02
330.00	122.01	0.04
335.00	121.93	-0.17
340.00	122.12	-0.16
345.00	122.38	-0.09
350.00	122.98	0.27
352.74	122.91	0.14

[a] Standard uncertainty u is $u(T)$ = 0.05 K, and the combined expanded uncertainty of the heat capacity is $U_c(C_{p,m}) = 0.01C_{p,m}$ (0.95 level of confidence). Values are reported with one digit more than is justified by the experimental uncertainty to avoid round-off errors in calculations based on these results.

[b] $\delta_{rel} = 100\times\left(C_{pm} - C^{o}_{pm}\right)/C^{o}_{pm}$, where heat capacity is calculated by means of Eqs. 1 and 2 with parameters from Table 2 in the main article.

Table S4. Experimental heat capacity of Ga_2Se_3 (in J K^{-1} mol^{-1}) obtained using the relaxation technique (Quantum Design PPMS)[a].

T / K	C_{pm} / J·K⁻¹·mol⁻¹	δ_{rel} [b]	*T*/ K	C_{pm} / J·K⁻¹·mol⁻¹	δ_{rel} [b]
	Run 1			Run 2	
302.342	122.89	1.76	302.439	122.95	1.80
295.330	121.75	1.11	295.426	121.94	1.26
288.354	121.44	1.17	288.434	121.47	1.19
281.395	120.83	1.00	281.448	120.90	1.06
274.418	120.66	1.22	274.466	120.74	1.29
267.450	119.14	0.34	267.491	119.22	0.41
260.483	118.11	-0.11	260.510	118.18	-0.05
253.487	117.72	0.02	253.540	117.79	0.08
246.508	117.08	-0.03	246.553	117.18	0.05
239.525	116.51	0.01	239.558	116.63	0.12
232.536	115.91	0.08	232.567	115.99	0.15
225.558	115.21	0.10	225.588	115.24	0.13
218.579	114.47	0.14	218.604	114.49	0.16
211.591	113.44	-0.02	211.618	113.45	-0.01
204.606	112.50	-0.04	204.630	112.48	-0.06
197.627	111.39	-0.14	197.639	111.46	-0.08
190.641	110.41	-0.05	190.653	110.44	-0.03
183.656	109.23	-0.06	183.656	109.37	0.07
176.670	108.04	0.03	176.661	108.08	0.07
169.683	106.71	0.10	169.683	106.77	0.16
162.704	105.08	0.02	162.692	105.18	0.12
155.722	102.96	-0.38	155.633	103.24	-0.08
148.738	101.11	-0.35	148.655	101.31	-0.13
141.724	98.945	-0.44	141.640	99.155	-0.20
134.704	96.657	-0.43	134.625	96.893	-0.15
127.718	94.306	-0.23	127.647	94.441	-0.05
120.738	91.527	-0.19	120.659	91.678	0.02
113.751	88.371	-0.22	113.667	88.529	0.00
106.769	84.980	-0.14	106.689	85.121	0.07
100.737	81.757	-0.06	100.641	81.995	0.30
95.708	79.086	0.32	95.607	79.260	0.62
90.650	76.219	0.77	90.570	76.302	0.95
85.602	72.762	0.71	85.517	72.850	0.92
80.545	68.937	0.41			
75.541	64.860	-0.08	75.437	64.926	0.15
70.461	60.756	-0.28	70.361	60.933	0.15
65.416	56.646	-0.25	65.337	56.670	-0.09
60.383	52.384	-0.19	60.331	52.309	-0.25
55.341	47.875	-0.26	55.266	47.898	-0.08
50.281	43.135	-0.46	50.219	43.121	-0.36
45.230	38.065	-1.08	45.165	38.056	-0.94
40.166	32.900	-1.12	40.106	32.868	-1.02
35.099	27.438	-0.61	35.063	27.362	-0.73
30.078	21.557	-0.10	30.028	21.515	-0.01
28.039	19.154	0.48	28.014	19.136	0.55
26.051	16.601	-0.04	25.994	16.692	0.93

T / K	C_{pm} / J·K^{-1}·mol^{-1}	δ_{rel} [b]	T/ K	C_{pm} / J·K^{-1}·mol^{-1}	δ_{rel} [b]
	Run 1			Run 2	
24.023	14.249	0.79	23.994	14.280	1.26
22.011	11.893	1.13	21.986	11.933	1.72
19.975	9.6244	1.55	19.962	9.5984	1.43
17.965	7.4102	0.41	17.955	7.3532	-0.23
15.893	5.3717	-0.37	15.890	5.3569	-0.59
13.892	3.6419	-1.30	13.894	3.6436	-1.30
11.884	2.2580	-0.91	11.882	2.2575	-0.88
9.849	1.2305	0.23	9.857	1.2291	-0.15
7.806	0.56162	-1.22	7.809	0.56201	-1.28
6.207	0.27650	1.69	6.204	0.27497	1.28
5.145	0.15159	0.18	5.146	0.15179	0.26
4.271	0.087567	1.38	4.273	0.087630	1.32
3.566	0.052969	0.17	3.563	0.052772	0.01
3.030	0.034901	-2.76	3.030	0.034943	-2.64
2.567	0.024012	-5.60	2.568	0.024039	-5.56
2.202	0.022155	15.27	2.203	0.022114	14.96
1.918	0.014312	-6.62	1.917	0.014308	-6.57

[a] Standard uncertainty of temperature is $u(T)$=0.004 K, and the combined expanded uncertainty of heat capacity $U_c(C_{pm})$ with 0.95 level of confidence (k=2) is $U_c(C_{pm})$=0.1 C_{pm} below 10 K; $U_c(C_{pm})$=0.03 C_{pm} in temperature range 10 to 40 K; $U_c(C_{pm})$=0.02 C_{pm} in temperature range 40 to 300 K. Values are reported with more digits than is justified by the experimental uncertainty to avoid round-off errors in calculations based on these results. Measurements are performed in vacuum (residual pressure p<10^{-4} Pa). Excluded points are printed in gray.

[b] $\delta_{rel} = 100\times\left(C_{pm} - C_{pm}^{o}\right) / C_{pm}^{o}$, where heat capacity is calculated by means of Eqs. 1 and 2 with parameters from Table 2 in the main article.

Table S5. Experimental heat capacity of Ga_2Te_3 (in J K^{-1} mol^{-1}) obtained using Tian–Calvet calorimetry (SETARAM μDSC IIIa)[a].

T / K	C_{pm} /J·K^{-1}·mol^{-1}	δ_{rel}[b]
270.51	123.67	-0.18
275.00	123.93	-0.16
280.00	124.19	-0.15
285.00	124.50	-0.09
290.00	124.82	-0.01
295.00	125.08	0.04
300.00	125.23	0.02
305.00	125.55	0.14
310.00	125.76	0.18
315.00	125.86	0.16
320.00	126.02	0.19
325.00	126.07	0.15
330.00	125.97	-0.01
335.00	126.02	-0.02
340.00	126.12	0.01
345.00	126.02	-0.11
350.00	126.12	-0.05
352.87	126.07	-0.10

[a] Standard uncertainty u is $u(T)$ = 0.05 K, and the combined expanded uncertainty of the heat capacity is $U_c(C_{p,m}) = 0.01C_{p,m}$ (0.95 level of confidence). Values are reported with one digit more than is justified by the experimental uncertainty to avoid round-off errors in calculations based on these results.

[b] $\delta_{rel} = 100\times\left(C_{pm} - C^{o}_{pm}\right)/C^{o}_{pm}$, where heat capacity is calculated by means of Eqs. 1 and 2 with parameters from Table 2 in the main article.

Table S6. Experimental heat capacity of Ga_2Te_3 (in J K^{-1} mol^{-1}) obtained using the relaxation technique (Quantum Design PPMS)[a].

T / K	C_{pm} / J·K^{-1}·mol^{-1}	δ_{rel} [b]	*T*/ K	C_{pm} / J·K^{-1}·mol^{-1}	δ_{rel} [b]
	Run 1			Run 2	
302.752	126.08	0.62	302.762	126.68	1.10
296.639	123.67	-1.13	296.315	124.91	-0.13
289.616	125.30	0.39	289.310	126.10	1.04
282.579	124.88	0.30	282.289	125.66	0.94
275.765	124.02	-0.12	275.436	124.73	0.46
260.768	124.20	0.71	260.776	124.34	0.82
254.737	122.44	-0.41	254.395	122.96	0.03
247.688	122.04	-0.34	247.364	122.53	0.08
240.676	121.65	-0.23	240.354	122.05	0.12
233.651	121.18	-0.16	233.334	121.60	0.21
226.604	120.64	-0.10	226.294	121.02	0.24
219.628	120.14	0.02	219.321	120.53	0.36
212.647	119.28	-0.13	212.261	119.66	0.22
205.497	118.53	-0.13	205.212	118.90	0.21
198.471	117.64	-0.21	198.193	118.02	0.14
191.407	116.81	-0.20	191.151	117.17	0.14
184.392	115.93	-0.18	184.135	116.33	0.20
177.315	115.18	0.04	177.068	115.53	0.37
170.236	114.14	0.08	169.994	114.48	0.41
163.253	112.84	-0.02	163.015	113.19	0.32
156.112	111.38	-0.13	155.892	111.75	0.24
149.199	109.84	-0.23	148.956	110.08	0.04
142.142	108.16	-0.27	141.898	108.48	0.08
135.098	106.24	-0.35	134.873	106.45	-0.10
128.103	104.01	-0.54	127.880	104.23	-0.26
121.108	101.61	-0.64	120.888	101.93	-0.26
114.045	99.161	-0.49	113.828	99.475	-0.09
107.047	96.365	-0.36	106.836	96.529	-0.09
100.951	93.572	-0.28	100.754	93.814	0.08
95.881	91.421	0.23	95.706	91.681	0.62
90.842	88.913	0.62	90.676	89.006	0.84
85.760	85.693	0.51	85.594	85.916	0.89
80.600	82.202	0.41	80.436	82.553	0.98
75.546	78.271	-0.04	75.387	78.413	0.29
70.495	74.238	-0.34	70.351	74.378	0.00
65.421	70.058	-0.49	65.276	70.238	-0.06
60.386	65.795	-0.44	60.248	65.852	-0.17
55.291	61.286	-0.24	55.157	61.368	0.10
50.188	56.091	-0.67	50.060	56.150	-0.34
45.013	50.661	-0.82	44.994	50.735	-0.63
39.901	44.958	-0.68	39.885	45.005	-0.53
34.839	38.717	-0.46	34.826	38.742	-0.35
29.787	31.812	-0.21	29.777	31.865	0.00
27.825	29.130	0.48	27.818	29.171	0.65
25.861	26.173	0.56	25.792	26.039	0.45
23.857	23.113	0.71	23.824	23.154	1.11

T / K	C_{pm} / J·K⁻¹·mol⁻¹	δ_{rel} [b]	T/ K	C_{pm} / J·K⁻¹·mol⁻¹	δ_{rel} [b]
	Run 1			Run 2	
21.899	20.114	0.96	21.843	19.974	0.70
19.789	17.178	2.99	19.742	16.774	1.00
17.760	13.346	-1.86	17.724	13.497	-0.35
15.788	10.553	-0.46	15.793	10.454	-1.47
13.718	7.4466	-1.18	13.720	7.4398	-1.31
11.726	4.8410	-0.50	11.726	4.8363	-0.59
9.735	2.7367	0.53	9.733	2.7359	0.57
7.323	1.0569	1.39	7.355	1.0861	2.63
5.943	0.50200	-0.60	5.947	0.50070	-1.09
4.932	0.26054	-1.39	4.933	0.26137	-1.15
4.101	0.13915	-0.84	4.093	0.13931	-0.06
3.494	0.082454	0.30	3.495	0.082547	0.32
2.950	0.047728	0.48	2.951	0.047764	0.45
2.518	0.028925	0.38	2.517	0.028924	0.50
2.175	0.018463	0.77	2.175	0.018458	0.75
1.911	0.012213	-1.12	1.911	0.012254	-0.79

[a] Standard uncertainty of temperature is $u(T)$=0.004 K, and the combined expanded uncertainty of heat capacity $U_c(C_{pm})$ with 0.95 level of confidence (k=2) is $U_c(C_{pm})$=0.1 C_{pm} below 10 K; $U_c(C_{pm})$=0.03 C_{pm} in temperature range 10 to 40 K; $U_c(C_{pm})$=0.02 C_{pm} in temperature range 40 to 300 K. Values are reported with more digits than is justified by the experimental uncertainty to avoid round-off errors in calculations based on these results. Measurements are performed in vacuum (residual pressure p<10^{-4} Pa). Excluded points are printed in gray (see text).

[b] $\delta_{rel} = 100\times\left(C_{pm} - C_{pm}^{o}\right) / C_{pm}^{o}$, where heat capacity is calculated by means of Eqs. 1 and 2 with parameters from Table 2 in the main article.

Table S7. Experimental heat capacity of In_2S_3 (in J K^{-1} mol^{-1}) obtained using Tian–Calvet calorimetry (SETARAM μDSC IIIa)[a].

T / K	C_{pm} /J·K^{-1}·mol^{-1}	δ_{rel}[b]
262.02	112.25	0.01
265.00	112.64	0.12
270.00	113.19	0.24
275.00	113.68	0.33
280.00	114.04	0.34
285.00	114.11	0.12
290.00	113.88	-0.32
295.00	113.78	-0.62
300.00	114.14	-0.49
305.00	114.69	-0.15
310.00	114.76	-0.22
315.00	114.99	-0.11
320.00	115.34	0.14
323.32	115.47	0.22

[a] Standard uncertainty u is $u(T)$ = 0.05 K, and the combined expanded uncertainty of the heat capacity is $U_c(C_{p,m}) = 0.01C_{p,m}$ (0.95 level of confidence). Values are reported with one digit more than is justified by the experimental uncertainty to avoid round-off errors in calculations based on these results.

[b] $\delta_{rel} = 100\times\left(C_{pm} - C^{o}_{pm}\right) / C^{o}_{pm}$, where heat capacity is calculated by means of Eqs. 1 and 2 with parameters from Table 2 in the main article.

Table S8. Experimental heat capacity of In_2S_3 (in J K^{-1} mol^{-1}) obtained using the relaxation technique (Quantum Design PPMS)[a].

T / K	C_{pm} / J·K^{-1}·mol^{-1}	δ_{rel} [b]	*T*/ K	C_{pm} / J·K^{-1}·mol^{-1}	δ_{rel} [b]
	Run 1			Run 2	
302.399	115.45	0.58	302.409	115.92	0.99
295.686	115.31	0.69	295.496	115.93	1.24
288.691	115.16	0.86	288.497	115.62	1.27
281.726	114.32	0.48	281.515	114.80	0.92
274.721	113.44	0.13	274.527	113.86	0.52
267.723	112.60	-0.12	267.536	112.91	0.17
260.759	111.68	-0.38	260.561	112.05	-0.05
253.757	110.91	-0.46	253.561	111.21	-0.17
246.803	110.16	-0.46	246.581	110.47	-0.15
239.797	109.39	-0.39	239.572	109.67	-0.11
232.786	108.67	-0.22	232.572	108.93	0.05
225.801	107.73	-0.18	225.588	108.04	0.14
218.797	106.79	-0.06	218.584	107.01	0.18
211.768	105.50	-0.20	211.578	105.78	0.10
204.781	104.26	-0.22	204.586	104.54	0.08
197.779	103.03	-0.16	197.596	103.29	0.13
190.754	101.80	-0.01	190.587	102.04	0.27
183.770	100.41	0.08	183.608	100.62	0.33
176.776	98.791	0.06	176.602	99.045	0.36
169.758	97.151	0.15	169.591	97.376	0.43
162.706	95.280	0.16	162.548	95.483	0.42
155.757	93.081	-0.04	155.583	93.241	0.19
148.735	90.651	-0.28	148.555	90.897	0.05
141.720	88.126	-0.43	141.531	88.304	-0.15
134.732	85.439	-0.52	134.543	85.621	-0.22
127.732	82.681	-0.41	127.548	82.808	-0.16
120.703	79.571	-0.38	120.518	79.764	-0.03
113.685	76.137	-0.40	113.512	76.306	-0.06
106.725	72.399	-0.45	106.543	72.503	-0.17
100.684	69.058	-0.26	100.501	69.136	0.01
95.623	66.226	0.17	95.461	66.307	0.45
90.572	63.206	0.60	90.414	63.242	0.82
85.494	59.735	0.58	85.330	59.783	0.85
80.397	56.013	0.35	80.240	56.030	0.59
75.379	52.090	-0.21	75.226	52.191	0.20
70.303	48.334	-0.26	70.158	48.345	-0.02
65.229	44.531	-0.29	65.094	44.535	-0.06
60.154	40.628	-0.46	60.017	40.623	-0.22
55.094	36.889	-0.16	54.965	36.880	0.09
50.076	32.984	-0.31	49.951	32.964	-0.08
44.993	28.933	-0.72	44.875	28.897	-0.53
39.938	24.959	-0.67	39.823	24.913	-0.49
34.841	20.850	-0.38	34.742	20.808	-0.19
29.766	16.626	0.11	29.677	16.575	0.26
27.777	14.961	0.46	27.738	14.955	0.65
25.742	13.209	0.56	25.704	13.205	0.78

T / K	C_{pm} / J·K⁻¹·mol⁻¹	δ_{rel} [b]	T/ K	C_{pm} / J·K⁻¹·mol⁻¹	δ_{rel} [b]
	Run 1			Run 2	
23.796	11.511	0.37	23.763	11.508	0.59
21.793	9.9241	1.43	21.798	9.8370	0.50
19.709	8.2902	2.53	19.793	8.1694	0.21
17.716	6.4600	-0.76	17.718	6.3906	-1.85
15.701	4.8254	-2.03	15.741	4.8455	-2.24
13.675	3.3178	-2.15	13.695	3.3462	-1.73
11.719	2.0966	0.35	11.718	2.0956	0.34
9.697	1.1098	3.14	9.697	1.1157	3.69
7.672	0.46411	2.09	7.678	0.46468	1.92
5.933	0.17332	-1.85	5.934	0.17343	-1.85
4.935	0.088380	-2.76	4.935	0.088329	-2.81
4.107	0.047492	-0.57	4.110	0.047588	-0.62
3.440	0.026579	0.47	3.436	0.026512	0.60
2.950	0.016443	1.29	2.951	0.016444	1.19
2.516	0.010167	1.81	2.516	0.010155	1.69
2.180	0.0065595	0.40	2.180	0.0065614	0.43
1.915	0.0044153	-1.63	1.912	0.0043963	-1.61

[a] Standard uncertainty of temperature is $u(T)$=0.004 K, and the combined expanded uncertainty of heat capacity $U_c(C_{pm})$ with 0.95 level of confidence (k=2) is $U_c(C_{pm})$=0.1 C_{pm} below 10 K; $U_c(C_{pm})$=0.03 C_{pm} in temperature range 10 to 40 K; $U_c(C_{pm})$=0.02 C_{pm} in temperature range 40 to 300 K. Values are reported with more digits than is justified by the experimental uncertainty to avoid round-off errors in calculations based on these results. Measurements are performed in vacuum (residual pressure p<10^{-4} Pa). Excluded points are printed in gray (see text). Experimental results from Quantum Design PPMS have been multiplied by a factor of 0.979 to agree with the more accurate SETARAM μDSC IIIa.

[b] $\delta_{rel} = 100\times\left(C_{pm} - C^{\circ}_{pm}\right) / C^{\circ}_{pm}$, where heat capacity is calculated by means of Eqs. 1 and 2 with parameters from Table 2 in the main article.

Table S9. Experimental heat capacity of In_2Se_3 (in J K^{-1} mol^{-1}) obtained using Tian–Calvet calorimetry (SETARAM μDSC IIIa)[a].

T / K	C_{pm} /J·K^{-1}·mol^{-1}	δ_{rel}[b]
262.04	121.44	-0.07
265.00	121.62	-0.09
270.00	121.95	-0.09
275.00	122.27	-0.09
280.00	122.51	-0.16
285.00	122.83	-0.14
290.00	123.16	-0.12
295.00	123.49	-0.09
300.00	123.81	-0.06
305.00	124.19	0.02
310.00	124.42	-0.02
315.00	124.65	-0.05
320.00	124.89	-0.08
325.00	125.17	-0.06
330.00	125.54	0.03
335.00	125.77	0.01
340.00	126.05	0.03
345.00	126.38	0.09
350.00	126.61	0.08

[a] Standard uncertainty u is $u(T)$ = 0.05 K, and the combined expanded uncertainty of the heat capacity is $U_c(C_{p,m}) = 0.01C_{p,m}$ (0.95 level of confidence). Values are reported with one digit more than is justified by the experimental uncertainty to avoid round-off errors in calculations based on these results.

[b] $\delta_{rel} = 100\times\left(C_{pm} - C^{o}_{pm}\right) / C^{o}_{pm}$, where heat capacity is calculated by means of Eqs. 1 and 2 with parameters from Table 2 in the main article.

Table S10. Experimental heat capacity of In_2Se_3 (in J K^{-1} mol^{-1}) obtained using the relaxation technique (Quantum Design PPMS)[a].

T / K	C_{pm} / J·K^{-1}·mol^{-1}	δ_{rel} [b]	*T*/ K	C_{pm} / J·K^{-1}·mol^{-1}	δ_{rel} [b]
	Run 1			Run 2	
301.758	114.42	-7.72	301.878	116.20	-6.29
295.376	115.84	-6.30	295.092	118.54	-4.10
288.354	115.62	-6.16	288.093	117.36	-4.74
281.411	116.00	-5.53	281.168	117.28	-4.47
274.496	116.83	-4.51	274.229	118.24	-3.35
267.532	117.21	-3.84	267.293	118.27	-2.96
260.579	117.52	-3.21	260.393	117.99	-2.81
253.640	117.34	-2.96	253.395	118.31	-2.15
246.664	117.10	-2.74	246.429	118.01	-1.97
239.677	117.13	-2.27	239.482	117.75	-1.74
232.686	116.42	-2.39	232.490	117.14	-1.77
225.744	116.01	-2.23	225.455	116.64	-1.68
218.773	115.29	-2.30	218.497	116.04	-1.64
211.795	114.93	-2.03	211.561	115.52	-1.50
204.771	114.77	-1.54	204.533	115.24	-1.11
197.827	113.86	-1.65	197.604	114.47	-1.10
190.838	113.42	-1.30	190.682	113.87	-0.89
183.833	112.71	-1.12	183.638	113.17	-0.69
176.843	112.19	-0.70	176.678	112.31	-0.57
169.899	110.75	-1.01	169.670	111.38	-0.41
162.876	109.92	-0.66	162.696	110.18	-0.40
156.032	108.23	-1.01	155.780	108.65	-0.58
149.025	106.88	-0.88	148.750	107.34	-0.39
142.155	105.45	-0.69	141.904	105.67	-0.42
135.201	103.70	-0.60	134.986	103.77	-0.47
128.223	101.78	-0.44	127.982	101.79	-0.36
121.191	99.597	-0.27	120.927	99.686	-0.08
114.151	97.004	-0.19	113.928	97.168	0.07
107.174	94.226	0.02	106.979	94.088	-0.03
101.141	91.313	-0.01	100.911	91.248	0.05
96.048	88.758	0.13	95.832	88.793	0.31
90.941	86.019	0.36	90.752	86.081	0.56
85.900	82.859	0.36	85.710	82.844	0.49
80.763	79.166	0.12	80.580	79.128	0.24
75.668	75.156	-0.20	75.522	75.071	-0.17
70.548	70.919	-0.41	70.385	70.870	-0.29
65.450	66.408	-0.58	65.301	66.349	-0.47
60.416	61.529	-0.90	60.257	61.511	-0.68
55.341	56.527	-0.79	55.190	56.473	-0.61
50.167	50.972	-0.81	50.065	50.875	-0.78
45.084	45.025	-1.12	44.973	44.904	-1.10
40.003	38.990	-0.88	39.901	38.867	-0.87
34.906	32.677	-0.46	34.797	32.619	-0.21
29.802	26.190	0.28	29.724	26.102	0.34
27.822	23.654	0.67	27.778	23.640	0.86
25.853	21.116	1.04	25.800	21.057	1.09

T / K	C_{pm} / J·K-1·mol-1	δ_{rel} [b]	T/ K	C_{pm} / J·K-1·mol-1	δ_{rel} [b]
	Run 1			Run 2	
23.897	18.555	1.13	23.837	18.468	1.09
21.780	15.961	2.07	21.804	15.914	1.57
19.691	13.500	3.47	19.759	13.391	1.99
17.619	10.637	0.72	17.639	10.649	0.60
15.773	8.3038	-1.03	15.758	8.3021	-0.84
13.684	6.0098	-0.26	13.690	5.9912	-0.67
11.702	3.9997	0.23	11.702	3.9955	0.12
9.696	2.3253	1.33	9.696	2.3251	1.32
7.657	1.0843	1.33	7.662	1.0855	1.22
5.930	0.44684	0.16	5.932	0.44707	0.10
4.932	0.23206	-1.06	4.932	0.23206	-1.06
4.101	0.12372	0.04	4.103	0.12395	0.05
3.434	0.068136	0.16	3.430	0.067907	0.21
2.946	0.040694	-1.44	2.947	0.040800	-1.29
2.511	0.025145	0.97	2.511	0.025112	0.84
2.172	0.015990	0.51	2.171	0.015977	0.57
1.909	0.010710	-0.37	1.910	0.010700	-0.62

[a] Standard uncertainty of temperature is $u(T)$=0.004 K, and the combined expanded uncertainty of heat capacity $U_c(C_{pm})$ with 0.95 level of confidence (k=2) is $U_c(C_{pm})$=0.1 C_{pm} below 10 K; $U_c(C_{pm})$=0.03 C_{pm} in temperature range 10 to 40 K; $U_c(C_{pm})$=0.02 C_{pm} in temperature range 40 to 300 K. Values are reported with more digits than is justified by the experimental uncertainty to avoid round-off errors in calculations based on these results. Measurements are performed in vacuum (residual pressure p<10^{-4} Pa). Excluded points are printed in gray (see text).

[b] $\delta_{rel} = 100\times\left(C_{pm} - C^{o}_{pm}\right) / C^{o}_{pm}$, where heat capacity is calculated by means of Eqs. 1 and 2 with parameters from Table 2 in the main article.

Table S11. Experimental heat capacity of In_2Te_3 (in J K^{-1} mol^{-1}) obtained using Tian–Calvet calorimetry (SETARAM μDSC IIIa)[a].

T / K	C_{pm} /J·K^{-1}·mol^{-1}	δ_{rel}[b]
261.76	122.92	-0.17
265.00	123.04	-0.21
270.00	123.28	-0.21
275.00	123.53	-0.20
280.00	123.71	-0.24
285.00	123.90	-0.26
290.00	124.14	-0.23
295.00	124.39	-0.19
300.00	124.63	-0.14
305.00	124.94	-0.03
310.00	125.12	-0.01
315.00	125.24	-0.03
320.00	125.37	-0.04
325.00	125.55	0.00
330.00	125.67	0.00
335.00	125.80	0.02
340.00	125.86	-0.01

[a] Standard uncertainty u is $u(T)$ = 0.05 K, and the combined expanded uncertainty of the heat capacity is $U_c(C_{p,m}) = 0.01C_{p,m}$ (0.95 level of confidence). Values are reported with one digit more than is justified by the experimental uncertainty to avoid round-off errors in calculations based on these results.

[b] $\delta_{rel} = 100 \times \left(C_{pm} - C^{o}_{pm} \right) / C^{o}_{pm}$, where heat capacity is calculated by means of Eqs. 1 and 2 with parameters from Table 2 in the main article.

Table S12. Experimental heat capacity of In_2Te_3 (in J K^{-1} mol^{-1}) obtained using the relaxation technique (Quantum Design PPMS)[a].

T / K	C_{pm} / J·K⁻¹·mol⁻¹	δ_{rel} [b]	*T*/ K	C_{pm} / J·K⁻¹·mol⁻¹	δ_{rel} [b]
	Run 1			Run 2	
302.848	124.93	0.03	302.847	125.51	0.48
296.853	124.16	-0.42	296.480	125.24	0.45
289.789	125.07	0.52	289.450	125.52	0.89
282.752	124.30	0.14	282.407	124.84	0.59
275.763	123.50	-0.26	275.413	124.03	0.19
268.731	122.99	-0.40	268.396	123.43	-0.03
261.689	122.79	-0.27	261.359	123.09	-0.01
254.649	122.32	-0.34	254.329	122.64	-0.07
247.653	121.98	-0.30	247.330	122.29	-0.03
240.608	121.68	-0.20	240.295	121.97	0.06
233.546	121.40	-0.06	233.241	121.74	0.24
226.569	121.00	0.00	226.254	121.31	0.27
219.546	120.63	0.11	219.241	120.90	0.35
212.493	120.00	0.02	212.202	120.27	0.26
205.494	119.36	-0.06	205.203	119.58	0.15
198.466	118.70	-0.13	198.178	118.95	0.10
191.428	118.05	-0.16	191.148	118.28	0.06
184.421	117.44	-0.13	184.148	117.69	0.11
177.315	116.97	0.07	177.053	117.14	0.24
170.198	116.22	0.10	169.935	116.49	0.35
163.124	115.24	-0.01	162.876	115.49	0.23
156.108	114.11	-0.16	155.862	114.35	0.07
149.141	113.00	-0.22	148.901	113.20	-0.01
142.104	111.79	-0.21	141.871	111.99	0.00
135.042	110.35	-0.27	134.807	110.55	-0.05
128.007	108.63	-0.40	127.788	108.81	-0.19
121.034	106.74	-0.49	120.822	106.96	-0.24
114.014	104.74	-0.43	113.805	104.97	-0.15
106.945	102.50	-0.26	106.751	102.66	-0.03
100.898	100.22	-0.17	100.703	100.38	0.07
95.834	98.341	0.18	95.662	98.491	0.41
90.728	96.202	0.55	90.571	96.312	0.75
85.675	93.456	0.53	85.516	93.599	0.78
80.573	90.242	0.32	80.395	90.405	0.62
75.555	86.707	-0.01	75.402	86.831	0.25
70.444	82.953	-0.19	70.301	83.046	0.05
65.339	79.126	-0.07	65.238	79.188	0.11
60.356	74.719	-0.37	60.222	74.805	-0.10
55.267	70.197	-0.20	55.141	70.241	0.03
50.151	64.830	-0.61	50.033	64.856	-0.38
45.062	59.083	-0.99	44.949	59.095	-0.75
40.021	53.193	-0.85	39.917	53.196	-0.61
34.937	46.710	-0.62	34.840	46.668	-0.43
29.850	39.619	-0.28	29.771	39.563	-0.12
27.866	36.797	0.21	27.832	36.762	0.25
25.804	33.737	0.71	25.773	33.751	0.90

T / K	C_{pm} / J·K^{-1}·mol^{-1}	δ_{rel} [b]	T/ K	C_{pm} / J·K^{-1}·mol^{-1}	δ_{rel} [b]
	Run 1			Run 2	
23.836	30.621	0.94	23.809	30.641	1.15
21.842	27.312	0.95	21.766	27.233	1.13
19.800	23.601	-0.14	19.760	23.794	0.97
17.724	20.806	3.71	17.798	20.309	0.58
15.784	16.395	-0.80	15.712	16.171	-1.35
13.732	12.405	-1.44	13.738	12.391	-1.65
11.743	8.6066	-1.86	11.740	8.6071	-1.79
9.730	5.3086	0.87	9.764	5.2767	-0.76
7.719	2.5340	0.46	7.723	2.5214	-0.21
5.948	1.0135	1.01	5.948	1.0134	1.00
4.944	0.50811	0.16	4.941	0.50626	0.02
4.109	0.25658	0.04	4.107	0.25640	0.15
3.491	0.14312	-0.09	3.494	0.14349	-0.14
2.953	0.080108	-0.62	2.953	0.080102	-0.63
2.521	0.047727	-0.05	2.521	0.047792	0.09
2.193	0.030565	0.19	2.192	0.030589	0.42
1.915	0.019909	-0.07	1.915	0.019918	-0.03

[a] Standard uncertainty of temperature is $u(T)$=0.004 K, and the combined expanded uncertainty of heat capacity $U_c(C_{pm})$ with 0.95 level of confidence (k=2) is $U_c(C_{pm})$=0.1 C_{pm} below 10 K; $U_c(C_{pm})$=0.03 C_{pm} in temperature range 10 to 40 K; $U_c(C_{pm})$=0.02 C_{pm} in temperature range 40 to 300 K. Values are reported with more digits than is justified by the experimental uncertainty to avoid round-off errors in calculations based on these results. Measurements are performed in vacuum (residual pressure p<10^{-4} Pa). Excluded points are printed in gray (see text). Experimental results from Quantum Design PPMS have been multiplied by a factor of 0.988 to agree with the more accurate SETARAM μDSC IIIa.

[b] $\delta_{rel} = 100\times\left(C_{pm} - C^{\circ}_{pm}\right) / C^{\circ}_{pm}$, where heat capacity is calculated by means of Eqs. 1 and 2 with parameters from Table 2 in the main article.

4. Tabulated thermodynamic functions

Standard thermodynamic functions were calculated using fundamental thermodynamic relationships (assuming residual entropy at 0 K for all studied sesqui-chalcogenides to be 0 J·K-1·mol-1) and heat capacities $C^{o}_{pm}(T)$ represented by Eqs 1 and 2 using parameters listed in Table 2:

$$S^{o}_{m}(T) = \int_{0}^{T} \frac{C^{o}_{pm}(T)}{T} \mathrm{d}T \quad \text{(S1)}$$

$$\Delta_0^T H^{o}_{m} = \int_{0}^{T} C^{o}_{pm}(T)\,\mathrm{d}T \quad \text{(S2)}$$

$$\Delta_0^T G^{o}_{m} = \Delta_0^T H^{o}_{m} - TS^{o}_{m}(T) \quad \text{(S3)}$$

Table S13. Standard Thermodynamic Functions of Ga_2S_3 at p = 0.1 MPa.[a]

T / K	C^{o}_{pm} / J·K-1·mol-1	S^{o}_{m} / J·K-1·mol-1	$\Delta_0^T H^{o}_{m}$ / kJ·mol-1	$\Delta_0^T G^{o}_{m}$ / kJ·mol-1
1[b]	4.211E-04	1.474E-04	1.094E-07	-3.799E-08
2	2.947E-03	1.049E-03	1.549E-06	-5.482E-07
3	9.268E-03	3.281E-03	7.264E-06	-2.578E-06
4	2.141E-02	7.442E-03	2.202E-05	-7.752E-06
5	4.198E-02	1.425E-02	5.288E-05	-1.834E-05
6	7.413E-02	2.454E-02	1.098E-04	-3.740E-05
7	1.219E-01	3.932E-02	2.063E-04	-6.891E-05
8	1.902E-01	5.980E-02	3.604E-04	-1.179E-04
9	2.849E-01	8.738E-02	5.955E-04	-1.909E-04
10	4.125E-01	1.237E-01	9.412E-04	-2.956E-04
11	5.790E-01	1.705E-01	1.433E-03	-4.417E-04
12	7.890E-01	2.295E-01	2.114E-03	-6.406E-04
13	1.045	3.025E-01	3.027E-03	-9.054E-04
14	1.347	3.906E-01	4.218E-03	-1.251E-03
15	1.698	4.953E-01	5.737E-03	-1.692E-03
16	2.097	6.173E-01	7.630E-03	-2.247E-03
17	2.543	7.576E-01	9.946E-03	-2.933E-03
18	3.036	9.166E-01	1.273E-02	-3.768E-03
19	3.574	1.095	1.603E-02	-4.773E-03
20	4.157	1.293	1.989E-02	-5.965E-03
25	7.695	2.583	4.913E-02	-1.545E-02
30	11.93	4.354	9.801E-02	-3.260E-02
35	16.36	6.526	1.687E-01	-5.965E-02
40	20.62	8.991	2.613E-01	-9.835E-02
45	24.59	11.65	3.745E-01	-1.499E-01
50	28.32	14.44	5.068E-01	-2.151E-01
55	31.96	17.31	6.575E-01	-2.944E-01
60	35.54	20.24	8.263E-01	-3.883E-01
65	39.05	23.23	1.013	-4.969E-01
70	42.48	26.25	1.217	-6.206E-01

T / K	C^{o}_{pm} / J·K⁻¹·mol⁻¹	S^{o}_{m} / J·K⁻¹·mol⁻¹	$\Delta_0^T H^{o}_{m}$ / kJ·mol⁻¹	$\Delta_0^T G^{o}_{m}$ / kJ·mol⁻¹
75	45.82	29.29	1.437	-7.594E-01
80	49.08	32.35	1.675	-9.135E-01
85	52.24	35.42	1.928	-1.083
90	55.30	38.50	2.197	-1.268
95	58.26	41.57	2.481	-1.468
100	61.11	44.63	2.779	-1.683
110	66.47	50.71	3.418	-2.160
120	71.42	56.71	4.107	-2.697
130	75.96	62.60	4.845	-3.294
140	80.14	68.39	5.625	-3.949
150	83.96	74.05	6.446	-4.661
160	87.46	79.58	7.304	-5.430
170	90.66	84.98	8.194	-6.252
180	93.57	90.25	9.116	-7.129
190	96.20	95.38	10.07	-8.057
200	98.59	100.4	11.04	-9.036
210	100.7	105.2	12.04	-10.06
220	102.7	110.0	13.05	-11.14
230	104.4	114.6	14.09	-12.26
240	106.0	119.1	15.14	-13.43
250	107.4	123.4	16.21	-14.64
260	108.7	127.6	17.29	-15.90
270	109.8	131.8	18.38	-17.20
273.15	110.2	133.0	18.73	-17.61
280	110.9	135.8	19.48	-18.53
290	111.9	139.7	20.60	-19.91
298.15	112.6	142.8	21.51	-21.06
300	112.8	143.5	21.72	-21.33
310	113.6	147.2	22.85	-22.78
320	114.3	150.8	23.99	-24.27
330	115.0	154.4	25.14	-25.80
340	115.7	157.8	26.29	-27.36
350	116.3	161.2	27.45	-28.95

[a] The combined expanded uncertainty of heat capacity $U_c(C_{pm})$ as well as of all calculated thermodynamic values (with 0.95 level of confidence, k=2) is $U_c(X)$=0.1 X below 10 K; $U_c(X)$=0.03 X in temperature range 10 to 40 K; $U_c(X)$=0.02 X in temperature range 40 to 260 K; $U_c(X)$=0.01 X in temperature range 260 to 350 K, where X represents the heat capacity or the thermodynamic property. Values are reported with one digit more than is justified by the experimental uncertainty to avoid round-off errors in calculations based on these results.

[b] Extrapolated values.

Table S14. Standard Thermodynamic Functions of Ga_2Se_3 at p = 0.1 MPa.[a.]

T / K	C^{o}_{pm} / J·K-1·mol-1	S^{o}_{m} / J·K-1·mol-1	$\Delta_0^T H^{o}_{m}$ / kJ·mol-1	$\Delta_0^T G^{o}_{m}$ / kJ·mol-1
1[b]	6.440E-03	4.848E-03	2.870E-06	-1.979E-06
2	1.638E-02	1.206E-02	1.382E-05	-1.029E-05
3	3.511E-02	2.182E-02	3.852E-05	-2.693E-05
4	7.174E-02	3.637E-02	8.996E-05	-5.550E-05
5	1.386E-01	5.889E-02	1.921E-04	-1.023E-04
6	2.443E-01	9.285E-02	3.800E-04	-1.771E-04
7	3.991E-01	1.414E-01	6.971E-04	-2.929E-04
8	6.162E-01	2.081E-01	1.199E-03	-4.660E-04
9	9.094E-01	2.968E-01	1.955E-03	-7.164E-04
10	1.292	4.116E-01	3.047E-03	-1.068E-03
11	1.772	5.564E-01	4.571E-03	-1.550E-03
12	2.351	7.347E-01	6.624E-03	-2.192E-03
13	3.022	9.487E-01	9.303E-03	-3.031E-03
14	3.775	1.200	1.270E-02	-4.102E-03
15	4.601	1.488	1.688E-02	-5.443E-03
16	5.489	1.813	2.192E-02	-7.090E-03
17	6.430	2.174	2.787E-02	-9.081E-03
18	7.415	2.569	3.479E-02	-1.145E-02
19	8.441	2.997	4.272E-02	-1.423E-02
20	9.505	3.457	5.169E-02	-1.745E-02
25	15.32	6.190	1.135E-01	-4.129E-02
30	21.48	9.527	2.055E-01	-8.036E-02
35	27.49	13.29	3.280E-01	-1.373E-01
40	33.09	17.34	4.797E-01	-2.138E-01
45	38.25	21.54	6.582E-01	-3.109E-01
50	43.07	25.82	8.616E-01	-4.293E-01
55	47.69	30.14	1.089	-5.691E-01
60	52.15	34.48	1.338	-7.307E-01
65	56.44	38.83	1.610	-9.140E-01
70	60.55	43.16	1.902	-1.119
75	64.49	47.48	2.215	-1.346
80	68.25	51.76	2.547	-1.594
85	71.83	56.00	2.897	-1.863
90	75.21	60.21	3.265	-2.154
95	78.40	64.36	3.649	-2.465
100	81.38	68.46	4.049	-2.797
110	86.75	76.47	4.890	-3.522
120	91.38	84.22	5.781	-4.326
130	95.38	91.70	6.716	-5.206
140	98.84	98.90	7.687	-6.159
150	101.8	105.8	8.691	-7.183
160	104.4	112.5	9.722	-8.274
170	106.7	118.9	10.78	-9.431
180	108.6	125.0	11.85	-10.65
190	110.4	131.0	12.95	-11.93
200	111.9	136.7	14.06	-13.27
210	113.3	142.1	15.19	-14.66
220	114.5	147.4	16.33	-16.11

T / K	C_{pm}^{o} / J·K^-1^·mol^-1^	S_{m}^{o} / J·K^-1^·mol^-1^	$\Delta_0^T H_{m}^{o}$ / kJ·mol^-1^	$\Delta_0^T G_{m}^{o}$ / kJ·mol^-1^
230	115.6	152.6	17.48	-17.61
240	116.5	157.5	18.64	-19.16
250	117.4	162.3	19.81	-20.76
260	118.2	166.9	20.99	-22.41
270	118.9	171.4	22.17	-24.10
273.15	119.1	172.8	22.55	-24.64
280	119.6	175.7	23.36	-25.83
290	120.1	179.9	24.56	-27.61
298.15	120.6	183.2	25.54	-29.09
300	120.7	184.0	25.77	-29.43
310	121.1	188.0	26.97	-31.29
320	121.6	191.8	28.19	-33.19
330	122.0	195.6	29.41	-35.13
340	122.3	199.2	30.63	-37.10
350	122.7	202.8	31.85	-39.11

[a] The combined expanded uncertainty of heat capacity $U_c(C_{pm})$ as well as of all calculated thermodynamic values (with 0.95 level of confidence, k=2) is $U_c(X)$=0.1 X below 10 K; $U_c(X)$=0.03 X in temperature range 10 to 40 K; $U_c(X)$=0.02 X in temperature range 40 to 260 K; $U_c(X)$=0.01 X in temperature range 260 to 350 K, where X represents the heat capacity or the thermodynamic property. Values are reported with one digit more than is justified by the experimental uncertainty to avoid round-off errors in calculations based on these results.

[b] Extrapolated values.

Table S15. Standard Thermodynamic Functions of Ga_2Te_3 at p = 0.1 MPa.[a]

T / K	C^{o}_{pm} / J·K⁻¹·mol⁻¹	S^{o}_{m} / J·K⁻¹·mol⁻¹	$\Delta_0^T H^{o}_{m}$ / kJ·mol⁻¹	$\Delta_0^T G^{o}_{m}$ / kJ·mol⁻¹
1[b]	1.795E-03	6.088E-04	4.547E-07	-1.540E-07
2	1.418E-02	4.742E-03	7.094E-06	-2.389E-06
3	5.012E-02	1.622E-02	3.659E-05	-1.207E-05
4	1.290E-01	4.013E-02	1.216E-04	-3.896E-05
5	2.771E-01	8.327E-02	3.176E-04	-9.873E-05
6	5.221E-01	1.538E-01	7.080E-04	-2.146E-04
7	8.919E-01	2.602E-01	1.403E-03	-4.182E-04
8	1.412	4.115E-01	2.542E-03	-7.500E-04
9	2.102	6.159E-01	4.284E-03	-1.259E-03
10	2.969	8.807E-01	6.805E-03	-2.002E-03
11	4.011	1.211	1.028E-02	-3.042E-03
12	5.207	1.611	1.488E-02	-4.447E-03
13	6.528	2.079	2.074E-02	-6.286E-03
14	7.942	2.614	2.797E-02	-8.627E-03
15	9.416	3.212	3.664E-02	-1.154E-02
16	10.92	3.867	4.681E-02	-1.507E-02
17	12.44	4.575	5.849E-02	-1.929E-02
18	13.96	5.329	7.169E-02	-2.424E-02
19	15.48	6.125	8.642E-02	-2.996E-02
20	17.00	6.958	1.027E-01	-3.650E-02
25	24.71	11.579	2.069E-01	-8.255E-02
30	32.19	16.752	3.494E-01	-1.532E-01
35	39.11	22.24	5.279E-01	-2.506E-01
40	45.38	27.88	7.394E-01	-3.759E-01
45	51.07	33.56	9.807E-01	-5.295E-01
50	56.28	39.21	1.249	-7.114E-01
55	61.16	44.81	1.543	-9.215E-01
60	65.74	50.33	1.860	-1.159
65	70.05	55.76	2.200	-1.425
70	74.10	61.10	2.560	-1.717
75	77.90	66.35	2.941	-2.036
80	81.46	71.49	3.339	-2.380
85	84.78	76.53	3.755	-2.750
90	87.87	81.46	4.186	-3.145
95	90.73	86.29	4.633	-3.565
100	93.36	91.01	5.093	-4.008
110	97.99	100.1	6.051	-4.964
120	101.9	108.8	7.051	-6.009
130	105.2	117.1	8.086	-7.139
140	107.9	125.0	9.152	-8.350
150	110.3	132.5	10.24	-9.639
160	112.3	139.7	11.36	-11.00
170	114.0	146.6	12.49	-12.43
180	115.5	153.2	13.64	-13.93
190	116.9	159.4	14.80	-15.49
200	118.1	165.5	15.97	-17.12
210	119.2	171.2	17.16	-18.80
220	120.2	176.8	18.36	-20.54

T / K	C^{o}_{pm} / J·K^{-1}·mol^{-1}	S^{o}_{m} / J·K^{-1}·mol^{-1}	$\Delta_0^T H^{o}_{m}$ / kJ·mol^{-1}	$\Delta_0^T G^{o}_{m}$ / kJ·mol^{-1}
230	121.1	182.2	19.56	-22.34
240	121.9	187.3	20.78	-24.19
250	122.6	192.3	22.00	-26.08
260	123.3	197.2	23.23	-28.03
270	123.9	201.8	24.46	-30.03
273.15	124.0	203.3	24.86	-30.67
280	124.4	206.3	25.71	-32.07
290	124.8	210.7	26.95	-34.15
298.15	125.1	214.2	27.97	-35.88
300	125.2	214.9	28.20	-36.28
310	125.5	219.1	29.46	-38.45
320	125.8	223.0	30.71	-40.66
330	126.0	226.9	31.97	-42.91
340	126.1	230.7	33.23	-45.20
350	126.2	234.3	34.49	-47.53

[a] The combined expanded uncertainty of heat capacity $U_c(C_{pm})$ as well as of all calculated thermodynamic values (with 0.95 level of confidence, k=2) is $U_c(X)$=0.1 X below 10 K; $U_c(X)$=0.03 X in temperature range 10 to 40 K; $U_c(X)$=0.02 X in temperature range 40 to 260 K; $U_c(X)$=0.01 X in temperature range 260 to 350 K, where X represents the heat capacity or the thermodynamic property. Values are reported with one digit more than is justified by the experimental uncertainty to avoid round-off errors in calculations based on these results.

[b] Extrapolated values.

Table S16. Standard Thermodynamic Functions of In_2S_3 at p = 0.1 MPa.[a]

T / K	C^{o}_{pm} / J·K^{-1}·mol^{-1}	S^{o}_{m} / J·K^{-1}·mol^{-1}	$\Delta_0^T H^{o}_{m}$ / kJ·mol^{-1}	$\Delta_0^T G^{o}_{m}$ / kJ·mol^{-1}
1[b]	7.210E-04	2.559E-04	1.892E-07	-6.669E-08
2	5.087E-03	1.797E-03	2.652E-06	-9.415E-07
3	1.711E-02	5.783E-03	1.288E-05	-4.469E-06
4	4.366E-02	1.388E-02	4.166E-05	-1.387E-05
5	9.523E-02	2.859E-02	1.085E-04	-3.443E-05
6	1.840E-01	5.313E-02	2.445E-04	-7.432E-05
7	3.239E-01	9.125E-02	4.935E-04	-1.452E-04
8	5.308E-01	1.472E-01	9.146E-04	-2.628E-04
9	8.196E-01	2.255E-01	1.582E-03	-4.470E-04
10	1.202	3.308E-01	2.585E-03	-7.227E-04
11	1.684	4.672E-01	4.020E-03	-1.119E-03
12	2.260	6.378E-01	5.985E-03	-1.669E-03
13	2.914	8.440E-01	8.566E-03	-2.406E-03
14	3.628	1.086	1.183E-02	-3.368E-03
15	4.382	1.362	1.584E-02	-4.589E-03
16	5.159	1.669	2.061E-02	-6.102E-03
17	5.945	2.006	2.616E-02	-7.937E-03
18	6.733	2.368	3.250E-02	-1.012E-02
19	7.522	2.753	3.962E-02	-1.268E-02
20	8.318	3.159	4.754E-02	-1.563E-02
25	12.50	5.459	9.947E-02	-3.700E-02
30	16.81	8.119	1.727E-01	-7.082E-02
35	21.06	11.03	2.675E-01	-1.186E-01
40	25.18	14.11	3.831E-01	-1.814E-01
45	29.15	17.31	5.190E-01	-2.599E-01
50	33.03	20.58	6.745E-01	-3.546E-01
55	36.88	23.91	8.492E-01	-4.659E-01
60	40.70	27.28	1.043	-5.938E-01
65	44.49	30.69	1.256	-7.387E-01
70	48.24	34.13	1.488	-9.008E-01
75	51.92	37.58	1.738	-1.080
80	55.53	41.05	2.007	-1.277
85	59.05	44.52	2.294	-1.491
90	62.45	47.99	2.597	-1.722
95	65.72	51.46	2.918	-1.970
100	68.83	54.91	3.254	-2.236
110	74.52	61.74	3.972	-2.820
120	79.55	68.44	4.742	-3.471
130	83.98	74.99	5.561	-4.188
140	87.88	81.36	6.420	-4.970
150	91.32	87.54	7.317	-5.814
160	94.37	93.53	8.245	-6.720
170	97.07	99.34	9.203	-7.685
180	99.48	105.0	10.19	-8.706
190	101.7	110.4	11.19	-9.783
200	103.6	115.7	12.22	-10.91
210	105.4	120.8	13.26	-12.10
220	107.1	125.7	14.33	-13.33

T / K	C^{o}_{pm} / J·K⁻¹·mol⁻¹	S^{o}_{m} / J·K⁻¹·mol⁻¹	$\Delta_0^T H^{o}_{m}$ / kJ·mol⁻¹	$\Delta_0^T G^{o}_{m}$ / kJ·mol⁻¹
230	108.5	130.5	15.40	-14.61
240	109.9	135.1	16.50	-15.94
250	111.0	139.7	17.60	-17.31
260	112.0	144.0	18.72	-18.73
270	112.9	148.3	19.84	-20.19
273.15	113.2	149.6	20.20	-20.66
280	113.7	152.4	20.97	-21.70
290	114.2	156.4	22.11	-23.24
298.15	114.6	159.6	23.05	-24.53
300	114.7	160.3	23.26	-24.82
310	115.0	164.0	24.41	-26.44
320	115.2	167.7	25.56	-28.10

[a] The combined expanded uncertainty of heat capacity $U_c(C_{pm})$ as well as of all calculated thermodynamic values (with 0.95 level of confidence, k=2) is $U_c(X)$=0.1 X below 10 K; $U_c(X)$=0.03 X in temperature range 10 to 40 K; $U_c(X)$=0.02 X in temperature range 40 to 260 K; $U_c(X)$=0.01 X in temperature range 260 to 320 K, where X represents the heat capacity or the thermodynamic property. Values are reported with one digit more than is justified by the experimental uncertainty to avoid round-off errors in calculations based on these results.

[b] Extrapolated values.

Table S17. Standard Thermodynamic Functions of In_2Se_3 at p = 0.1 MPa.[a]

T / K	C^{o}_{pm} / J·K-1·mol-1	S^{o}_{m} / J·K-1·mol-1	$\Delta_0^T H^{o}_{m}$ / kJ·mol-1	$\Delta_0^T G^{o}_{m}$ / kJ·mol-1
1[b]	1.588E-03	5.425E-04	4.045E-07	-1.379E-07
2	1.238E-02	4.163E-03	6.218E-06	-2.109E-06
3	4.377E-02	1.418E-02	3.195E-05	-1.058E-05
4	1.136E-01	3.514E-02	1.065E-04	-3.409E-05
5	2.460E-01	7.332E-02	2.800E-04	-8.659E-05
6	4.647E-01	1.360E-01	6.273E-04	-1.889E-04
7	7.902E-01	2.306E-01	1.245E-03	-3.693E-04
8	1.238	3.640E-01	2.249E-03	-6.631E-04
9	1.816	5.419E-01	3.765E-03	-1.112E-03
10	2.523	7.687E-01	5.924E-03	-1.763E-03
11	3.348	1.047	8.850E-03	-2.667E-03
12	4.277	1.378	1.266E-02	-3.875E-03
13	5.292	1.759	1.743E-02	-5.439E-03
14	6.372	2.191	2.326E-02	-7.410E-03
15	7.499	2.669	3.019E-02	-9.836E-03
16	8.655	3.189	3.827E-02	-1.276E-02
17	9.829	3.749	4.751E-02	-1.623E-02
18	11.01	4.345	5.793E-02	-2.027E-02
19	12.21	4.972	6.954E-02	-2.493E-02
20	13.42	5.629	8.236E-02	-3.023E-02
25	19.78	9.300	1.652E-01	-6.730E-02
30	26.38	13.49	2.806E-01	-1.241E-01
35	32.95	18.05	4.289E-01	-2.028E-01
40	39.33	22.87	6.098E-01	-3.050E-01
45	45.44	27.86	8.218E-01	-4.318E-01
50	51.20	32.95	1.064	-5.838E-01
55	56.62	38.08	1.333	-7.613E-01
60	61.68	43.23	1.629	-9.646E-01
65	66.39	48.35	1.950	-1.194
70	70.75	53.44	2.293	-1.448
75	74.79	58.46	2.657	-1.728
80	78.53	63.41	3.040	-2.033
85	81.97	68.27	3.441	-2.362
90	85.14	73.05	3.859	-2.715
95	88.06	77.73	4.292	-3.092
100	90.74	82.32	4.739	-3.492
110	95.46	91.19	5.671	-4.360
120	99.43	99.67	6.646	-5.315
130	102.8	107.8	7.658	-6.352
140	105.6	115.5	8.700	-7.469
150	108.1	122.9	9.769	-8.661
160	110.1	129.9	10.86	-9.925
170	111.9	136.6	11.97	-11.26
180	113.4	143.1	13.10	-12.66
190	114.8	149.3	14.24	-14.12
200	116.0	155.2	15.39	-15.64
210	117.1	160.9	16.56	-17.22
220	118.1	166.3	17.74	-18.86

T / K	C^{o}_{pm} / J·K^{-1}·mol^{-1}	S^{o}_{m} / J·K^{-1}·mol^{-1}	$\Delta_0^T H^{o}_{m}$ / kJ·mol^{-1}	$\Delta_0^T G^{o}_{m}$ / kJ·mol^{-1}
230	119.0	171.6	18.92	-20.55
240	119.9	176.7	20.12	-22.29
250	120.7	181.6	21.32	-24.08
260	121.4	186.3	22.53	-25.92
270	122.1	190.9	23.75	-27.81
273.15	122.3	192.4	24.13	-28.41
280	122.7	195.4	24.97	-29.74
290	123.3	199.7	26.20	-31.71
298.15	123.8	203.1	27.21	-33.36
300	123.9	203.9	27.44	-33.73
310	124.4	208.0	28.68	-35.79
320	125.0	211.9	29.92	-37.89
330	125.5	215.8	31.18	-40.03
340	126.0	219.5	32.43	-42.21

[a] The combined expanded uncertainty of heat capacity $U_c(C_{pm})$ as well as of all calculated thermodynamic values (with 0.95 level of confidence, k=2) is $U_c(X)$=0.1 X below 10 K; $U_c(X)$=0.03 X in temperature range 10 to 40 K; $U_c(X)$=0.02 X in temperature range 40 to 260 K; $U_c(X)$=0.01 X in temperature range 260 to 340 K, where X represents the heat capacity or the thermodynamic property. Values are reported with one digit more than is justified by the experimental uncertainty to avoid round-off errors in calculations based on these results.

[b] Extrapolated values.

Table S18. Standard Thermodynamic Functions of In_2Te_3 at p = 0.1 MPa.[a.]

T / K	C^{o}_{pm} / J·K-1·mol-1	S^{o}_{m} / J·K-1·mol-1	$\Delta_0^T H^{o}_{m}$ / kJ·mol-1	$\Delta_0^T G^{o}_{m}$ / kJ·mol-1
1[b]	2.791E-03	9.426E-04	7.045E-07	-2.381E-07
2	2.282E-02	7.491E-03	1.124E-05	-3.740E-06
3	8.502E-02	2.651E-02	6.019E-05	-1.934E-05
4	2.326E-01	6.846E-02	2.095E-04	-6.434E-05
5	5.290E-01	1.488E-01	5.751E-04	-1.690E-04
6	1.036	2.865E-01	1.338E-03	-3.811E-04
7	1.800	5.000E-01	2.733E-03	-7.672E-04
8	2.844	8.054E-01	5.031E-03	-1.412E-03
9	4.157	1.214	8.511E-03	-2.412E-03
10	5.699	1.730	1.342E-02	-3.875E-03
11	7.414	2.352	1.997E-02	-5.907E-03
12	9.251	3.075	2.829E-02	-8.613E-03
13	11.16	3.891	3.849E-02	-1.209E-02
14	13.11	4.789	5.063E-02	-1.642E-02
15	15.04	5.760	6.470E-02	-2.169E-02
16	16.93	6.791	8.069E-02	-2.796E-02
17	18.77	7.873	9.855E-02	-3.529E-02
18	20.55	8.996	1.182E-01	-4.372E-02
19	22.28	10.15	1.396E-01	-5.329E-02
20	23.97	11.34	1.628E-01	-6.404E-02
25	32.22	17.58	3.034E-01	-1.362E-01
30	39.95	24.15	4.841E-01	-2.404E-01
35	47.09	30.85	7.019E-01	-3.779E-01
40	53.63	37.57	9.539E-01	-5.489E-01
45	59.60	44.24	1.237	-7.535E-01
50	65.07	50.81	1.549	-9.911E-01
55	70.08	57.25	1.887	-1.261
60	74.68	63.54	2.249	-1.563
65	78.91	69.69	2.633	-1.897
70	82.78	75.68	3.038	-2.260
75	86.34	81.52	3.461	-2.653
80	89.60	87.19	3.901	-3.075
85	92.58	92.72	4.356	-3.525
90	95.30	98.09	4.826	-4.002
95	97.78	103.3	5.309	-4.505
100	100.0	108.4	5.803	-5.035
110	103.9	118.1	6.823	-6.168
120	107.0	127.3	7.878	-7.395
130	109.5	135.9	8.961	-8.711
140	111.6	144.1	10.07	-10.11
150	113.4	151.9	11.19	-11.59
160	114.8	159.3	12.33	-13.15
170	116.1	166.3	13.49	-14.78
180	117.2	172.9	14.66	-16.47
190	118.1	179.3	15.83	-18.23
200	119.0	185.4	17.02	-20.06
210	119.8	191.2	18.21	-21.94
220	120.5	196.8	19.41	-23.88

T / K	C^{o}_{pm} / J·K⁻¹·mol⁻¹	S^{o}_{m} / J·K⁻¹·mol⁻¹	$\Delta_0^T H^{o}_{m}$ / kJ·mol⁻¹	$\Delta_0^T G^{o}_{m}$ / kJ·mol⁻¹
230	121.2	202.2	20.62	-25.88
240	121.9	207.3	21.84	-27.92
250	122.5	212.3	23.06	-30.02
260	123.0	217.1	24.29	-32.17
270	123.6	221.8	25.52	-34.37
273.15	123.7	223.2	25.91	-35.07
280	124.0	226.3	26.76	-36.61
290	124.4	230.7	28.00	-38.89
298.15	124.7	234.1	29.02	-40.78
300	124.8	234.9	29.25	-41.22
310	125.1	239.0	30.50	-43.59
320	125.4	243.0	31.75	-46.00
330	125.7	246.8	33.00	-48.45
340	125.9	250.6	34.26	-50.93

[a] The combined expanded uncertainty of heat capacity $U_c(C_{pm})$ as well as of all calculated thermodynamic values (with 0.95 level of confidence, k=2) is $U_c(X)$=0.1 X below 10 K; $U_c(X)$=0.03 X in temperature range 10 to 40 K; $U_c(X)$=0.02 X in temperature range 40 to 260 K; $U_c(X)$=0.01 X in temperature range 260 to 340 K, where X represents the heat capacity or the thermodynamic property. Values are reported with one digit more than is justified by the experimental uncertainty to avoid round-off errors in calculations based on these results.

[b] Extrapolated values.
